# Nowcasting officially reported highly pathogenic avian influenza outbreak counts under heterogeneous reporting delays

**DOI:** 10.3389/fvets.2026.1958869

**Published:** 2026-10-08

**Authors:** Kun Xiang, Xiangbo Zhu

**Affiliations:** 1 Research Center for Machine Learning and Intelligent Surveillance, China Three Gorges University, Yichang, Hubei, China; 2 Plant Protection Research Center, Shenzhen Polytechnic University, Shenzhen, China

**Keywords:** African swine fever, HPAI, nowcasting, reporting delay, right truncation, surveillance, WAHIS

## Abstract

Official animal disease series are often incomplete due to delays in reporting. This retrospective observational study evaluated whether increased model complexity enhances probabilistic nowcasts of eventual officially reported outbreak-equivalent counts. We reconstructed historical information states from a fixed extraction from the World Organisation for Animal Health (WOAH) World Animal Health Information System (WAHIS), which included 46,860 highly pathogenic avian influenza (HPAI) and 72,690 African swine fever (ASF) outbreaks. Twelve prespecified methods were assessed over 78 locked cutoffs for 2024–2025 using the Weighted Interval Score (WIS), calibration diagnostics, block bootstrap, simulation, and a separately frozen USDA APHIS transfer evaluation. The primary HPAI analysis included 35,880 observations from 115 country–host units. At week closure, only 7.6% of eventual HPAI and 6.7% of ASF outbreak equivalents were visible, with completeness varying by host, region, year, season, and workload. The prespecified primary hypothesis was not supported: the delay-aware temporal network scored 6.9% worse than the hierarchical Bayesian comparator, and the relational graph changed WIS by only 0.4%. Gradient-boosted quantile regression improved WIS by 18.5% (absolute difference −0.0397, 95% interval −0.0553 to −0.0255), but the improvement reached 26.0% among high-volume reporters and reversed to a 6.3% deficit among units with minimal reporting history. Conditioning on the partial count was 24.7% worse than a matched no-delay network at cohort age 0 and performed better thereafter. The slower-reporting ASF system reversed the pooled contrast, whereas the APHIS module improved WIS by 10.1% after system-specific retraining. Adding an explicit mass at zero enhanced the transparent comparator by 18.8% and resulted in no detectable difference from gradient boosting; the neural model remained 17.4% worse under the same recalibration. In exploratory analyses, gradient boosting cleared the prespecified 5% gate under all six cohort definitions and all three maturity horizons. Performance deteriorated markedly for high-volume countries and regions excluded from training. These estimates pertain to eventual officially reported outbreak equivalents under the surveillance conditions studied, not undetected infections.

## 1 Introduction

Decisions about surveillance intensity, movement control, and resource allocation for transboundary animal diseases depend on the official record available at the time. For highly pathogenic avian influenza (HPAI) and African swine fever (ASF), that record is maintained in the World Animal Health Information System (WAHIS) by the World Organisation for Animal Health (WOAH). An outbreak reaches WAHIS only after detection, sampling, laboratory confirmation, submission by a national delegate, and validation (1–3). Each step takes time, and the delay varies by country, host, and workload (4). The record at the end of a week therefore contains only events reported by that time, rather than all events eventually attributed to the week. The most recent weeks are consequently right-truncated: in the extraction analyzed here, only 7.6% of the HPAI outbreak-equivalents eventually attributed to an occurrence week were present when that week closed, compared with 6.7% for ASF. An apparent downturn at the end of a curve can therefore reflect fewer events, slower reporting, or both, and a completed database snapshot cannot distinguish between these explanations. Analyses based on such snapshots face two additional problems. Random record-level splits can place the same outbreak chain, adjacent weeks, and neighboring countries in both training and test sets (5, 6), while an absent record can be interpreted as evidence of absent disease (7). Both problems remain relevant in veterinary machine learning, where risk mapping from completed snapshots is more common than prediction at a historical cutoff (8–10).

Machinery for handling delayed observations is well established outside veterinary surveillance. Reporting triangles and chain-ladder development factors originate in actuarial reserving (11); Bayesian nowcasting and time-varying reporting-probability models have been applied to dengue, influenza-like illness, and COVID-19 (12–17); and temporal and graph neural networks have drawn strength from multiple regions (18, 19). Recent benchmarks in applied machine learning have compared classical time-series models against deep sequence models for epidemic prediction and assembled multi-source frameworks intended for routine public health use (20, 21). That body of work motivates several of the architectures tested here, and it already reports the kind of conditional result our design is built to detect: across New Zealand and five partner countries, a recurrent network was the most accurate model in most comparisons, while a simple autoregressive model proved more stable across time frames (21). Which architecture wins thus depends on the data regime rather than capacity alone. Whether this machinery pays for itself in official animal disease reporting remains an open question: WAHIS timeliness has been characterized descriptively (4), but measuring notification intervals does not yield a predictive distribution and cannot correct a curve. The question is whether higher-capacity nowcasting variants produce a reproducible gain over simpler delay-aware methods once transferred to a setting where the reporting institution changes with the country, country–host–week counts are mostly zero, and delay tails reach the 99th percentile of 229 days in HPAI and 382 days in ASF. This comparison concerns scored predictive distributions rather than point accuracy (22, 23).

The estimand must distinguish delayed reporting from permanent non-reporting. WAHIS contains official reports, so an outbreak that is never detected, confirmed, or notified leaves no record that a database-based estimator could recover (24). Because delay correction and recovery of permanently unreported events are different problems, and only the first has an observable ground truth, we defined the target as officially reported outbreak equivalents filed within a 52-week maturity horizon. A simulation in which a known fraction of events was deleted before reporting illustrates what this target does and does not cover (Figure 1). Within this scope, four questions were specified before the test data were opened. The primary question was whether delay-aware temporal or relational neural models improved probabilistic accuracy over transparent delay-correction methods by at least a study-defined 5% relative WIS margin, the threshold selected to justify the added implementation and governance burden. The three supporting questions examined the completeness of recent HPAI and ASF curves at successive information cutoffs, variation in the reporting-delay distribution by host, region, year, season, and workload, and the transfer of the delay component to ASF and to a separately frozen US Department of Agriculture Animal and Plant Health Inspection Service (USDA APHIS) wild-bird detection system after system-specific retraining. We evaluated these questions using a fixed historical snapshot, weekly rolling-origin replay, one locked out-of-time test, matched ablations, ten simulation scenarios, and a multilevel block bootstrap. The HPAI prioritization rule was assessed separately as an operational outcome. The primary complexity hypothesis was not supported; the transfer of the delay component depended on the surveillance system; and the advantage of the method that cleared the gate was concentrated among units with the highest reporting volumes.

**Figure 1:**
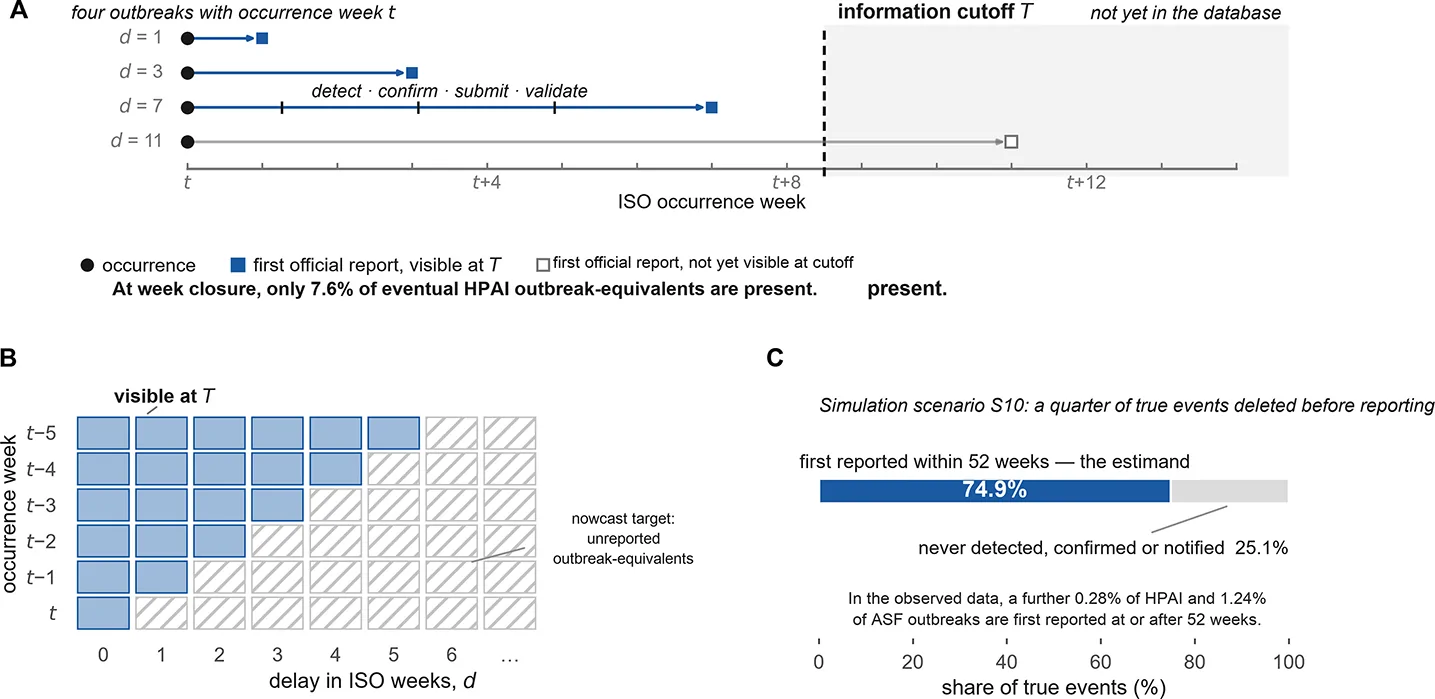
The observable reporting process and the estimand. **(A)** Four outbreaks with the same occurrence week t reach the official database after delays d that vary by country, host, and workload; at the information cutoff T, only reports dated on or before T are visible. **(B)** The reporting triangle at T: shaded cells are visible, hatched cells are outbreak equivalents not yet reported and constitute the nowcast target. **(C)** The identifiability boundary, measured in simulation scenario S10, in which a quarter of true events are deleted before reporting. The estimand covers 74.9% of true events whose first report is filed within 52 weeks; the deleted events lie outside it by construction and are unrecoverable from the database by any method. That scenario generates no reports beyond 52 weeks, so the further exclusion imposed by the horizon is annotated from the observed data rather than from the simulation.

## 2 Materials and methods

### 2.1 . Study design and estimand

This retrospective observational prediction study reconstructed historical information states and used rolling-origin evaluation. The unit of analysis was country × host stratum × ISO occurrence week for WAHIS and state × ISO occurrence week for APHIS. For unit *i* and occurrence week *t*, the target was the number of outbreak-equivalent units first reported in delay weeks 0–51 after onset. At cutoff *T*, the visible count included only first reports dated on or before *T*; the nowcast target was the unreported remainder (Figures 1A, B). Events first reported at or after 52 weeks, or never entered into the official system, lay outside the estimand (Figure 1C). Delay was defined by the difference between the ISO-week indices of onset and first report, not by elapsed days divided by seven. An outbreak beginning on the Sunday that closes week *t* and reported the following Monday is not visible at the cutoff for week *t*, although its day-based delay rounds to zero weeks. A day-based definition would therefore admit future reports into the age-0 visible count for 40.9% of HPAI and 41.4% of ASF outbreaks. ISO-week indexing makes “reported on or before *T*” equivalent to “delay ≤ cohort age.” Calendar-day delays were used only for descriptive summaries.

### 2.2 Data sources, eligibility, and sample

The primary data source was a fixed extraction of WOAH WAHIS immediate-notification and follow-up reports covering 1 January 2005 to 2 July 2026 (1). Analyses began in 2017 to align with the current official HPAI labels. Eligibility was determined using the exact English labels for poultry HPAI, non-poultry HPAI, and ASF; bovine HPAI was excluded. Selection operated at the label level because one English disease name can correspond to several identifiers. The complete identifier inventory is summarized in the Supplementary material, and the exclusion cascade is shown in Supplementary Figure S1. Hosts were categorized into wild and domestic (non-wild) strata, and the exclusion of mammalian hosts was prespecified as a sensitivity analysis. The extraction contained 60,958 HPAI-eligible long rows representing 46,860 unique outbreak identifiers and 76,264 ASF-eligible rows representing 72,690 identifiers. After restricting the data to 2017 onward, 38,602 HPAI and 68,440 ASF outbreaks remained. Repeated follow-up rows contributed report dates but did not create new outbreaks. Declared clusters were expanded by the reported number of outbreaks for the primary target; one-per-identifier and cluster-exclusion definitions were assessed in sensitivity analyses. Reconstructed reporting-triangle totals were required to equal eligible outbreak-equivalent totals. The full WAHIS panel comprised 180 countries and two host strata. The primary active cohort required at least two mature outbreak equivalents during development and contained 115 HPAI and 54 ASF country–host units. The locked HPAI evaluation included 115 units × 78 cutoffs × four cohort ages (35,880 observations); the ASF evaluation included 16,848 observations. All eligible records and active units were used. Sensitivity analyses repeated scoring at six development-period cohort thresholds, from the complete 360-unit panel to the 38 highest-volume reporters (Supplementary Table S11).

The APHIS transfer evaluation used official wild-bird HPAI detection records from the United States Department of Agriculture Animal and Plant Health Inspection Service (25), accessed on 2 August 2026. Of 19,544 records, 19,140 had a known collection date and a non-negative delay of fewer than 52 weeks; these records yielded 50 active states, 31 mature 2025 cutoffs, and 6,200 observations. APHIS counts detection records rather than WAHIS outbreak equivalents.

### 2.3 Historical information states, predictors, and methods compared

First-report date was the earliest reporting date associated with an outbreak identifier. Weekly cutoffs were set at the end of each ISO week (Sunday, UTC), and every method used only reports dated on or before the cutoff. Because the source did not preserve historical revisions of onset date, the final onset date was carried across reconstructed versions; report-date visibility was replayed exactly, but onset-date revision history was not. Mature outcomes required 52 weeks of follow-up. Negative delays and delays of 52 weeks or more were excluded from the target and retained for data-quality auditing. Completeness was estimated at each cutoff from an expanding pool of mature occurrence weeks. The WAHIS implementation excluded the week maturing exactly at the cutoff, whereas the stated rule and APHIS implementation included it; neither convention uses future reports, and the aligned refit is reported in Supplementary Table S13. Predictors included lagged own-unit and adjacent-host counts, global recent load, week-of-year harmonics, a linear time trend, host, WOAH region, and country metadata. Geographic adjacency and centroid neighbors came from Natural Earth (26); directed live-animal trade edges used the 2015–2021 mean from the Food and Agriculture Organization of the United Nations (FAO) FAOSTAT database (27). Livestock stocks lagged by 2 years were examined but omitted from the primary model because they varied little within countries. Graph and cross-host relations were lagged by 1 week before message passing. All trailing-window features supplied to an incidence encoder ended before the target week. Including the target week would use its observed count once in the incidence prior and again in the reporting update, artificially narrowing the posterior. The exclusion was checked on every row; the target week's observed count entered the neural models only through the closed-form reporting update.

Twelve prespecified methods were evaluated on identical cutoffs (Table 1). B4, the strongest transparent delay-aware method on HPAI validation, served as the reference for the primary complexity gate. B8 was the primary neural model, while B9 incorporated a relational graph. B6 and B7 were matched no-delay versions of B8 and B9, allowing each pair to isolate the contribution of the reporting layer. Complete configurations and the validation-stage architecture comparison are provided in Supplementary Tables S1, S2. B4 modeled log incidence on mature cohorts using a Poisson generalized linear model that included global, region, country, host, seasonal, trend, and recent-history terms. Country effects utilized a ridge penalty (λ = 1), and a country received its own effect only after at least five mature outbreak equivalents. A discrete delay hazard indexed by cohort age × region × host was re-estimated at each cutoff and combined with incidence through a Gamma–Poisson predictive update. B3 employed a penalized maximum-likelihood Laplace fit of a NobBS-style random-walk incidence and a shared Dirichlet delay distribution within each region–host series, as the published sampler requires JAGS (13). B8 combined a two-layer causal transformer (28), a discrete-time reporting hazard, and the Gamma–Poisson update; B9 added a relation-aware graph block (29) to account for geographic, trade, and cross-host relations. Deep ensembles propagated within- and between-member variance (30); ensemble sizes, seeds, and training settings are detailed in Supplementary Table S1. Delay-aware predictions were constrained to be no lower than the count already reported. The matched no-delay variants remained unconstrained, ensuring that the ablations preserved their intended specifications.

**Table 1:** The twelve prespecified methods and an exploratory two-part count model.

Method	What it does	Delay	Role in the design
B0 raw observed	Visible count at the cutoff, uncorrected	No	Naive floor
B1 completeness ratio	Inflation by a global, region–host or country–host ratio (three variants)	Fixed	Transparent
B2 chain ladder	Development factors on the reporting triangle	Fixed	Transparent
B3 NobBS-style	Random-walk log incidence with a shared Dirichlet delay, Laplace fit	Yes	Transparent; ASF gate reference
B4 hierarchical Bayes	Poisson GLM incidence with ridge-shrunk country effects, age × region × host hazard, Gamma–Poisson	Yes	Transparent; HPAI gate reference
B5 gradient boosting	XGBoost quantile regression on the unreported remainder (Section 2.3.1)	Yes, modeled directly	Best-scoring prespecified method
B6 temporal, no delay	Causal transformer, reporting layer removed	No	Matched ablation for B8
B7 temporal+graph, no delay	B6 plus the relational graph block	No	Matched ablation for B9
B8 delay-aware temporal	B6 plus discrete reporting hazard and Gamma–Poisson update	Yes	Primary neural variant
B9 delay-aware temporal+graph	B8 plus the relational graph block	Yes	Graph ablation
Z1 boosted hurdle-NB^a^	Logistic zero part plus Poisson-objective positive part with negative-binomial dispersion, features and tree settings matched to B5	Yes, modeled directly	Exploratory zero-inflation analysis

“Fixed” denotes a completeness correction that does not vary by host, region, year, season, or reporting load. B4 and B3 were the strongest transparent comparators for HPAI and ASF validation, respectively, and became the references for the 5% complexity gate for each disease.

^a^Fitted after completion of the locked evaluation. Z1 is exploratory and does not enter the prespecified complexity gate.

#### 2.3.1 Configuration of the gradient-boosting method (B5)

B5 estimated the unreported remainder $U_{it}^{\left(T\right)}=Y_{it}^{\left(52\right)}-c_{it}^{\left(T\right)}$ rather than the eventual total. The observed count was added after prediction, maintaining the predictive lower bound at or above what had already been reported. Since the label was the remainder, the observed count could also be used once as a conditioning predictor without duplicating it in an incidence prior. Seven XGBoost models estimated the frozen quantile grid using pinball loss (31, 32). An eighth Poisson-objective model estimated the predictive mean μ; the dispersion implied by the fitted 80% interval was used for the reported predictive variance. Quantiles were floored at zero and made monotonic by a running maximum before scoring. Continuous quantile regression does not inherently provide the event probability needed for operational analysis. B5, therefore, utilized


$$\begin{array}{l} Pr\left(U>0\right)=\frac{\mu }{1+\;\mu }, \end{array}$$

the probability of a positive count under a geometric distribution with mean μ. The interval-derived dispersion did not enter this calculation. The monotone transform preserves B5′s ranking, but it is not a probability fitted directly to the event; its Brier score was interpreted accordingly. All eight boosters shared one validation-selected hyperparameter set and a fixed random seed. Models were refitted quarterly on expanding windows of mature cohorts. Exact tree settings, the seed, and the validation search are reported in Supplementary Tables S1, S18. The 29 predictors comprised observed count, cohort age, and as-of completeness; own-unit and adjacent-host counts lagged by 1, 2, 4, 8, 13, and 26 weeks; own-unit window sums over 4, 13, 26, and 52 weeks; two global-load window sums; four cross-host, geographic, and trade-neighbor aggregates; week-of-year sine and cosine terms; a linear year trend; and a wild-host indicator. Every predictor was constructed only from information available by the cutoff.

### 2.4 Development, freezing, and locked evaluation

WAHIS data through 2021 were used for development, 2022–2023 for validation, and 78 weekly cutoffs from 7 January 2024 to 29 June 2025 for the locked test. Training windows expanded, and models were refitted quarterly. Hyperparameters were selected based on validation macro-WIS with a calibration penalty. The selection score was WIS × [1 + |Cov80–0.80| + |Cov95–0.95|], with ties broken in favor of fewer parameters. Eligibility, preprocessing, the active cohort, hyperparameters, and the analysis protocol were frozen before test outcomes were examined; all methods were then run together, and the test was used once for scoring. The APHIS evaluation was frozen separately, with development through 2023, validation in 2024, and 31 mature cutoffs from January to August 2025. Architecture, hyperparameters, and evaluation rules were transferred without graph search or post-test recalibration, while model weights were retrained on APHIS development data. It therefore evaluates transfer after system-specific retraining rather than a fixed fitted model. ASF used the WAHIS design and frozen architecture as a cross-disease stress test under a different host mix and a longer reporting-delay distribution.

### 2.5 Outcomes, statistical analysis, and sensitivity analyses

All methods emitted quantiles at 0.025, 0.1, 0.25, 0.5, 0.75, 0.9, and 0.975, from which 50%, 80%, and 95% intervals were formed. The primary outcome was the macro-averaged Weighted Interval Score (WIS) across active units, pooled over cohort ages 0–3; lower scores are better (22, 23). Secondary outcomes were mean absolute error, bias, interval coverage and width, randomized probability integral transforms for count distributions (33), positive-target WIS, age-specific performance, and performance by development-period reporting volume (0–1, 2–8, 9–49, and ≥50 outbreak equivalents for HPAI). Operational outcomes were recall and the number needed to investigate under fixed weekly review budgets, and the Brier score for the event that at least one outbreak equivalent remained unreported (34). Each method supplied that probability from its predictive distribution except B5, whose construction is described in Section 2.3.1. The study used the complete eligible frozen extraction, every mature locked cutoff, and every development-defined active unit. Sample size was therefore determined by the surveillance systems rather than recruitment, and no prospective power calculation was performed.

Because no field-standard minimal important difference exists for WIS in veterinary nowcasting, a 5% relative improvement was prespecified as a model-adoption rule rather than as a biological threshold. Sensitivity analyses also applied 2.5% and 10% gates. Operational importance was assessed separately through fixed-budget recall and the number needed to investigate. Model differences were paired within cutoff and evaluated with 1,000 cutoff-block bootstrap resamples (35). Prespecified robustness analyses used 2,000 resamples with cutoff, country, and crossed country-by-cutoff pigeonhole blocks; a Bonferroni-adjusted 99% interval covered five secondary contrasts against the common baseline. WAHIS intervals for the matched delay-module contrasts were computed after the test with the same 1,000-resample procedure and treated as exploratory. Coverage was reported as an estimate. Nested discrete-time reporting-hazard models added host, region, year, season, and recent load sequentially and were compared using likelihood-ratio χ^2^ statistics, deviance, and AIC.

Ten known-truth simulation scenarios, each with 100 replicates, covered stable and changing delays, reporting heterogeneity and backlog, sparse counts, a long delay tail, graph misspecification, cold-start units, and permanent non-reporting (Supplementary Table S14; Supplementary Figure S2). Sensitivity analyses varied cluster handling, target definition, bootstrap blocking, and disease inclusion. Target-changing analyses, including mammalian-host exclusion, rebuilt the target and refitted models; cohort-threshold analyses only re-aggregated frozen predictions. Graph inference used the matched B8–B9 comparison; fine-grained graph runs with a different model dimension were excluded from comparative inference. Spatial extrapolation was assessed by refitting after holding out six high-volume countries and each of the four WOAH regions.

Revise the caption accordingly; do not call 38,602 and 68,440 target records because the >=52-week tail has not yet been removed from those counts.

#### 2.5.1 Additional calibration and robustness analyses

Four analyses were specified after the completion of the locked evaluation and treated as exploratory; none entered the adoption gate or altered the frozen B0–B9 predictions. They comprised a newly fitted two-part model, recalibration of stored predictive distributions, re-aggregation under alternative active-cohort rules, and reconstruction and refitting under alternative maturity horizons. The two-part boosted model Z1 used a hurdle formulation (36, 37): a logistic booster estimated Pr(*U* > 0), a Poisson-objective booster estimated E(*U* – 1|*U* > 0), and a negative-binomial dispersion was fitted by moments on positive training rows. Features and tree settings matched B5. For B4 and B8, the negative-binomial distribution recovered from each stored mean and variance was mixed with an explicit point mass at zero, *U*_*ZI*_~πδ_0_+(1−π)*NB*(*r, p*). One weight π per cohort age was estimated on validation data by matching the observed share of zero remainders and then applied unchanged to the locked test. This was recalibration of stored predictions rather than model refitting.

Active-cohort sensitivity used development-period thresholds of 0, 1, 2, 5, 10, and 50 mature outbreak equivalents. Because cohort membership only affected which units were scored, frozen predictions were re-aggregated without refitting. Maturity-horizon sensitivity compared 26, 52, and 78 weeks. The reporting tensor was rebuilt to 78 delay bins, and B4, B5, and Z1 were refitted at every cutoff; neural models were not refitted for this analysis. Horizons were compared over the common 53-cutoff window from 7 January 2024 to 5 January 2025, during which age-0 targets had matured under all three definitions. Analyses used Python 3.11. Reporting followed TRIPOD+AI, PROBAST+AI, and STROBE-Vet principles (38–40).

## 3 Results

### 3.1 Reporting delay and its heterogeneity

All conservation checks passed (Table 2). Among 38,726 post-2017 HPAI and 68,603 post-2017 ASF records entering the descriptive delay audit, 32 (0.083%) and 155 (0.226%), respectively, had negative first-report delays and were excluded before target construction. Among the 38,602 HPAI and 68,440 ASF records retained before tail exclusion, 107 (0.277%) and 846 (1.236%) were first reported at or after 52 weeks and therefore lay outside the target. The median delay was 14 days for both diseases, but the distributions diverged in the tail: the 90th and 99th percentiles were 56 and 229 days for HPAI, compared with 122 and 382 days for ASF. Locations were flagged as approximate for 44.8% of HPAI rows and 12.8% of ASF rows. Figure 1 shows the observable reporting process and the estimand. At age 0, only 7.6% of eventual HPAI and 6.7% of ASF outbreak equivalents were visible; completeness reached 78.2% and 70.5% at 4 weeks, and 90.1% and 79.1% at 8 weeks (Figure 2A). These pooled estimates combine distinct reporting pathways. For ASF, the 90th percentile of first-report delay was 235 days in domestic hosts and 77.8 days in wild hosts; for HPAI, the median was 11 days in domestic hosts and 17 days in wild hosts (Figure 2B). Reporting hazards were also non-stationary. From the delay-only model to the full model, AIC fell by 5,682.4 for HPAI and 9,815.6 for ASF, and every sequential addition of host, region, year, season, and recent load reduced deviance in both diseases. The weakest single step was the ASF calendar year (Δdeviance 24.15, 1 df, *p* = 8.9 × 10^−7^). The AIC changes and separated curves (Figures 2C, D), rather than the *p*-values alone, support adaptive estimation of completeness instead of one pooled curve.

**Table 2:** Source records, retained target records, and first-report delay audit.

Source	Raw	Eligible before tail exclusion	0–51-week target	Median (d)	P90 (d)	P99 (d)	Negative delay n/N (%)	>=52 weeks n/N (%)
HPAI (WAHIS)	46,860	38,602	38,495	14	56	229	32/38,726 (0.083%)	107/38,602 (0.277%)
ASF (WAHIS)	72,690	68,440	67,594	14	122	382	155/68,603 (0.226%)	846/68,440 (1.236%)
HPAI wild birds (APHIS)	19,544	19,259	19,140	–	-	–	10/19,544 (0.052%)	119/19,544 (0.609%)

“Raw” counts source entities after disease-label selection but before applying the remaining exclusion criteria; d, first-report delay in days for WAHIS. APHIS rows are detection records; 0.609% combines 119 records at or beyond 52 weeks among 19,544 raw records. –, not used in the primary descriptive comparison.

**Figure 2:**
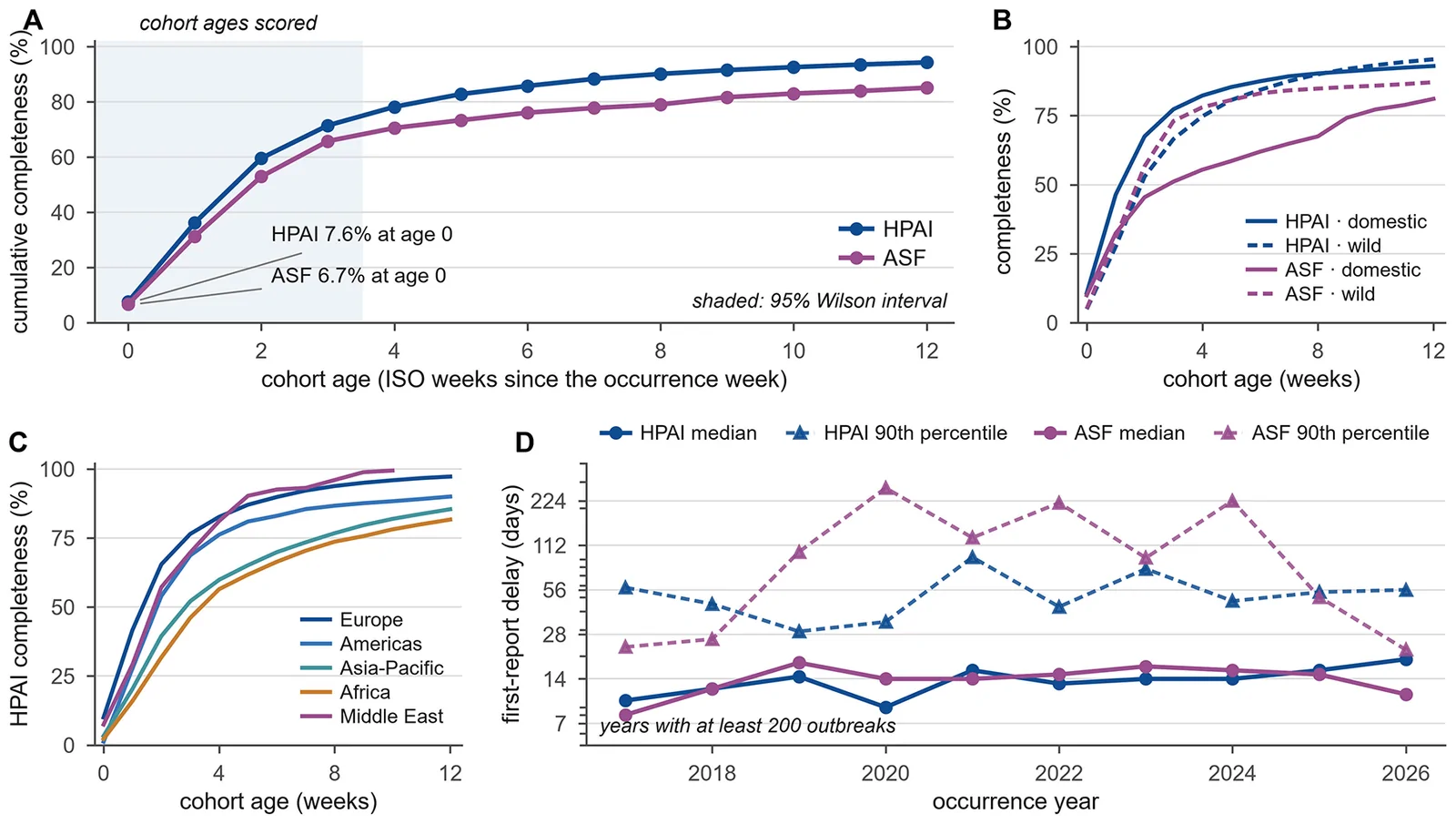
Reporting delay is heterogeneous and non-stationary. **(A)** Global cumulative completeness by cohort age with 95% Wilson intervals; the shaded band marks the four prespecified scored ages. **(B)** Host-specific completeness, domestic vs. wild. **(C)** HPAI completeness by WOAH region, focusing on the five largest regions. **(D)** Median and 90th-percentile first-report delay by occurrence year, for years with at least 200 outbreaks; note the logarithmic vertical scale.

### 3.2 The locked HPAI test and the complexity gate

The prespecified hypothesis was not supported. The delay-aware temporal network (B8) had a pooled macro-WIS of 0.2294, compared with 0.2145 for the transparent hierarchical Bayesian comparator (B4), making it 6.9% worse (Table 3; Figure 3A). B8 failed the prespecified gate, which required a 5% improvement, and its point estimate favored B4. The evidence did not support the stronger claim that B8 was consistently worse because that conclusion depended on the bootstrap blocking scheme. Resampling cutoffs provided a 95% interval of 0.0045 to 0.0259 for the absolute WIS difference, excluding zero, while resampling whole countries yielded an interval of −0.0123 to 0.0582, which included zero. The matched graph model (B9) scored 0.2284, 0.4% lower than B8 but still 6.5% worse than B4. The graph had already failed validation, where it improved WIS in only one of six encoder-by-seed configurations (Supplementary Table S2); the locked test confirmed that validation-stage decision.

**Table 3:** Selected HPAI locked-test results, primary active cohort, cohort ages 0–3 pooled.

Model	WIS	vs B4	MAE	Cov80	Cov95	Cov80+	Bias
B4 hierarchical Bayes	0.2145	–	0.3296	97.1%	98.6%	79.1%	0.1782
B5 gradient boosting	0.1748	+18.5%	0.2589	93.4%	97.5%	70.8%	0.0275
B6 temporal, no delay	0.2762	−28.8%	0.4118	97.9%	99.2%	–	0.0943
B8 delay-aware temporal	0.2294	−6.9%	0.3390	95.6%	98.0%	68.2%	0.0556
B9 delay-aware temporal+graph	0.2284	−6.5%	0.3363	95.7%	98.0%	–	0.0516
Exploratory analyses; excluded from the prespecified complexity gate							
Z1 boosted hurdle-NB	0.1939	+9.6%	0.2728	97.0%	98.6%	78.6%	0.0202
B4 with an explicit zero mass	0.1741	+18.8%	0.2807	97.5%	98.9%	82.1%	0.0346
B8 with an explicit zero mass	0.2044	+4.7%	0.2963	96.6%	98.4%	75.2%	−0.0203

Macro-averaged across 115 active country–host units and 78 cutoffs (*n* = 35,880). Lower WIS and MAE indicate better performance. “vs B4” represents the relative WIS improvement over the frozen transparent hierarchical Bayesian comparator; positive values favor the listed model. Cov80+ is 80% interval coverage restricted to the 4,981 cells with a positive mature target, where every frozen model under-covers. The three exploratory rows were produced after completion of the locked evaluation and do not enter any gate decision: Z1 is a model newly fitted on the same design, while the two zero-mass rows recalibrate the frozen predictive distributions with one mixing weight per cohort age fitted on validation data (Section 3.3.2).

**Figure 3:**
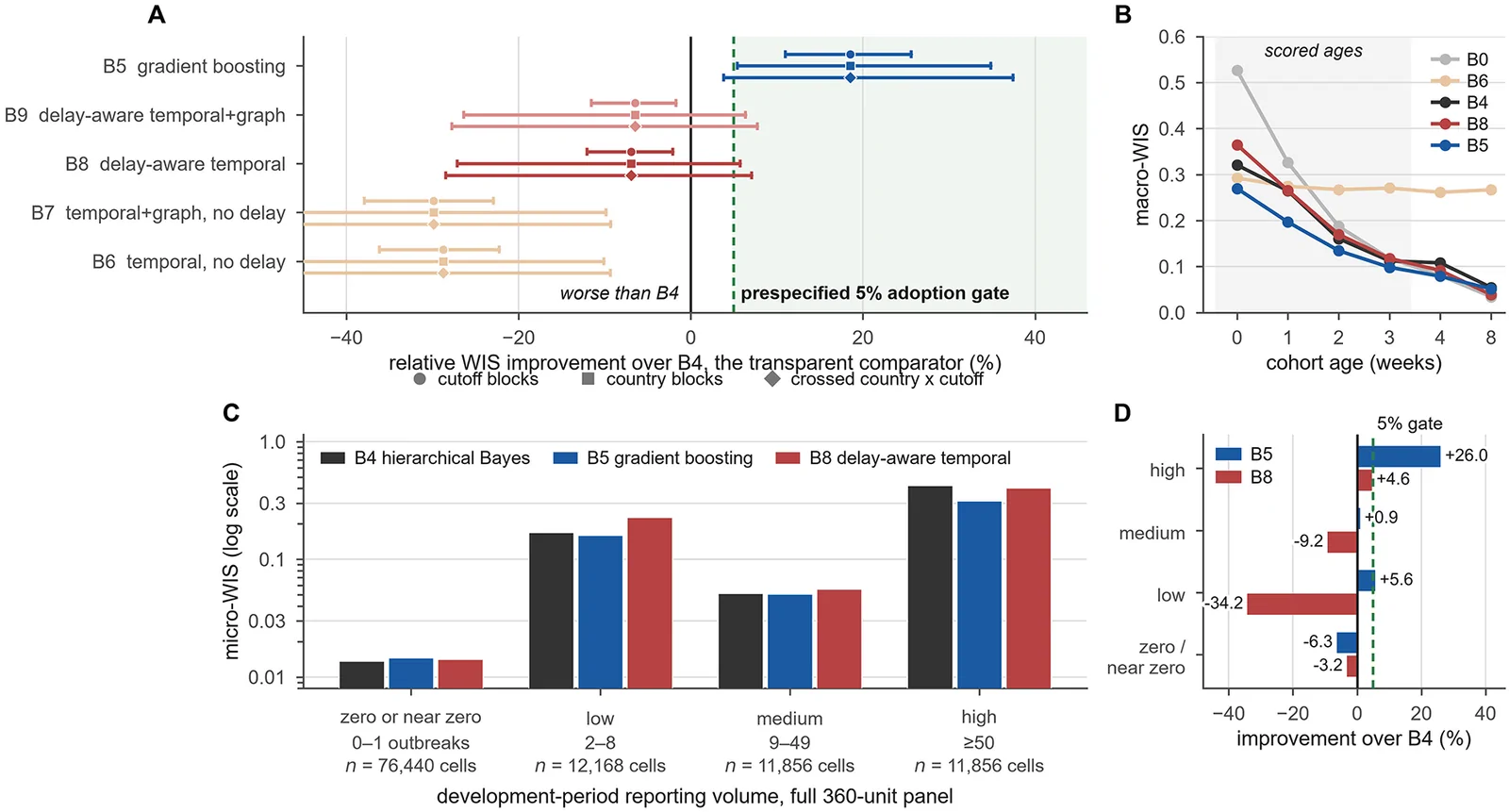
The locked HPAI test and where the advantage lies. **(A)** Relative WIS improvement over the transparent comparator B4 for five methods, each with 95% paired bootstrap intervals under three blockings; the dashed line represents the prespecified 5% adoption gate, and the shaded region indicates the acceptance zone. Only gradient boosting lies entirely beyond the gate, and it does so under all three blockings. **(B)** Macro-WIS by cohort age; the delay-aware network crosses the matched no-delay ablation between ages 0 and 1. **(C)** Micro-WIS by development-period reporting volume on the full 360-unit panel, logarithmic scale. **(D)** The same comparison as a relative improvement over B4, showing that the gradient-boosting advantage is concentrated among high-volume reporters and reverses where units report almost nothing. *n* = 35,880 scored cells in **(A, B)**; 112,320 in **(C, D)**.

Gradient-boosted quantile regression on the unreported remainder (B5, configured as described in Section 2.3.1) was the only prespecified method to clear the gate. It scored 0.1748, an 18.5% improvement over B4, with a paired absolute WIS difference of −0.0397 (95% cutoff-block interval −0.0553 to −0.0255). The result was robust to a Bonferroni-adjusted 99% interval for the five contrasts against B4 (−0.0602 to −0.0203), country-level blocking (−0.0748 to −0.0116), and crossed country-by-cutoff blocking (−0.0803 to −0.0082). B5 also had a lower MAE than B4 (0.2589 vs. 0.3296). Thus, the tested gradient-boosting formulation improved predictive accuracy, while the tested deep relational architecture did not.

#### 3.2.1 Performance by reporting volume

The 18.5% pooled improvement was concentrated among units with high reporting volumes. In the separate full 360-unit panel used for the prespecified reporting-volume-stratum analysis, B5 scored 0.3135 against 0.4236 for B4 in the high-volume stratum, resulting in a 26.0% gap. The gap was 5.6% at low volume and 0.9% at medium volume (0.0508 vs. 0.0513). In the zero-or-near-zero stratum, which contains 76,440 of the 112,320 stratified observations, B4 was the better model (0.0137 vs. 0.0145, a 6.3% deficit for B5). The gain is therefore concentrated among high-volume units; for units with almost no reporting history, the transparent model is better (Figures 3C, D). The deep model degrades in the same direction but further: B8 was 4.6% better than B4 among high-volume reporters, 9.2% worse at medium volume, and 34.2% worse at low volume, so its pooled deficit is driven by the sparser units rather than being spread evenly across the panel. Within the balanced primary active cohort, macro and micro averages coincide to floating-point precision because every unit contributes the same number of cutoff–age cells, but that does not neutralize volume, since the per-cell score still scales with count; what reveals the dependence is the full-panel stratified breakdown, not the primary macro average, and for that reason, the conclusions of this study are stated for units with established reporting history rather than for WAHIS members in general.

### 3.3 Calibration and the origin of the gradient-boosting advantage

Calibration needs the same treatment as accuracy. Overall, 80% and 95% coverage was 93.4% and 97.5% for B5, and 97.1% and 98.6% for B4. However, 30,899 of the 35,880 scored cells had a zero mature target, and 92.2% of the cells had an eventual total exactly equal to the already-observed count—a figure that the zero-width raw-observed baseline attains trivially. Overall coverage on this panel is therefore largely a property of scoring a sparse count panel, rather than evidence of calibration, and the prespecified 0.75–0.85 band for 80% intervals was not attained by the evaluated models and is difficult to interpret here. When restricted to the 4,981 cells with a positive mature target, all representative models undercover: 70.8% and 88.5% for B5, 79.1% and 90.2% for B4, and 68.2% and 85.3% for B8 (Figure 4A). Positive-target WIS ranks the models in the same order as the pooled score (1.017 for B5, 1.353 for B4, 1.545 for B8), so the ordering does not depend on the zero-dominated summary. Despite its lower WIS, B5 had lower positive-target coverage than B4; accuracy and interval coverage therefore support different aspects of the comparison.

**Figure 4:**
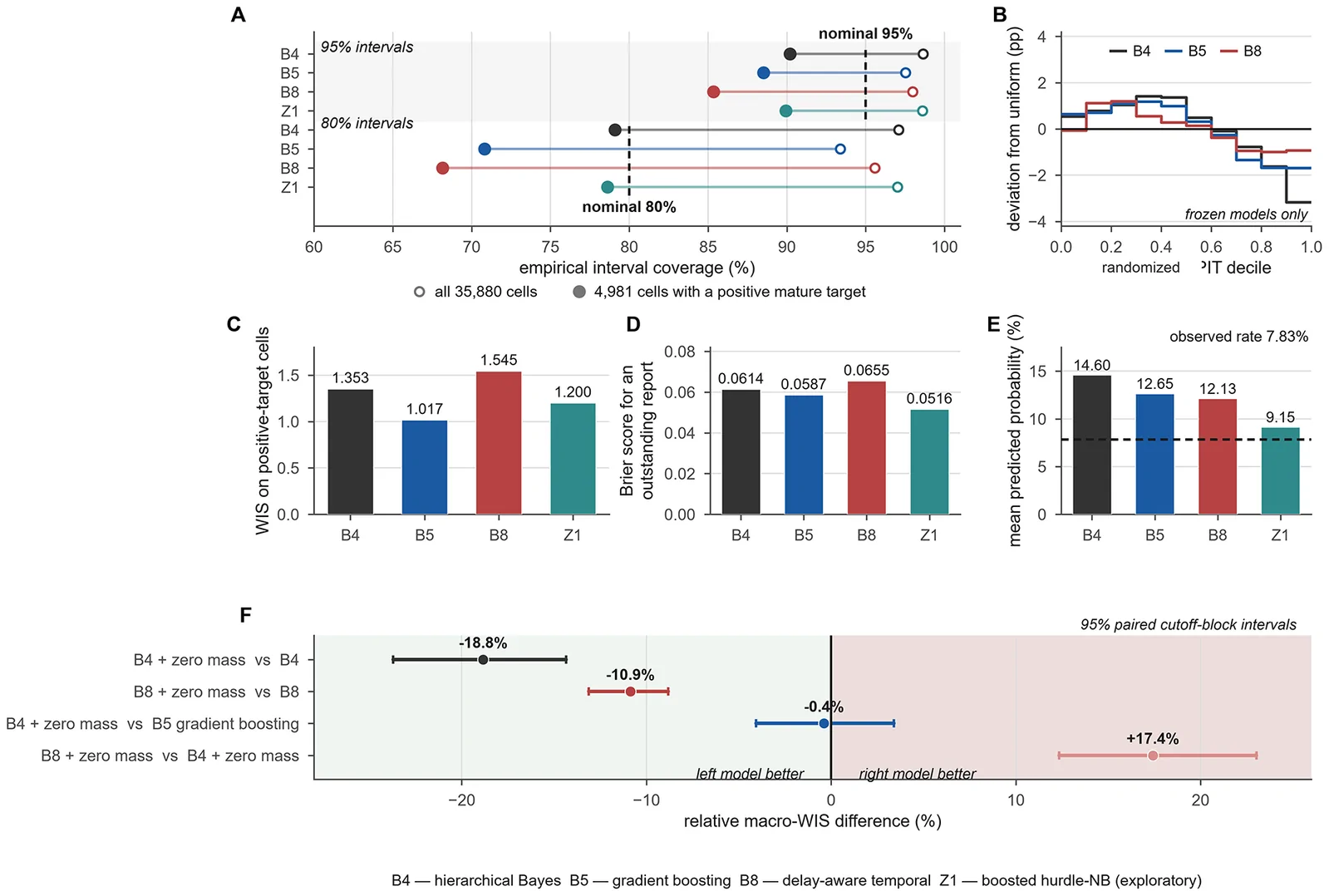
Calibration under a zero-dominated target and an exploratory two-part count model. **(A)** Empirical coverage of nominal 80% and 95% intervals, across all 35,880 scored cells (open markers) and the 4,981 cells with a positive mature target (filled markers). Overall coverage is largely influenced by structural zeros; every model undercovers where the target is positive, and the two-part model Z1 recovers most of what quantile regression loses. **(B)** Randomized probability integral transforms, plotted as deviations from uniformity in percentage points, for the three frozen models for which they were computed. **(C)** WIS restricted to positive-target cells. **(D)** Brier score for the event that at least one outbreak-equivalent remains unreported. **(E)** Mean predicted probability of that event compared to its observed rate of 7.83%. **(F)** What happens when the two frozen delay-aware models are given an explicit mass at zero, with one mixing weight per cohort age fitted on validation data and applied unchanged to the locked test. Both improve, after which the comparison no longer distinguishes the transparent comparator from gradient boosting, whereas the neural model remains far behind. Z1 and the zero-mass variants are exploratory and do not enter any gate decision.

The randomized probability integral transforms point the same way and separate calibration from accuracy. Against the 0.100 expected under uniformity, the top decile holds 0.068 of B4′s transforms and 0.083 of B5′s, with a matching excess in the lower bins—the signature of mild over-prediction, consistent with their positive bias (0.1782 and 0.0275). The raw observed curve shows the mirror image, 0.153 in the top decile, as an estimator that never adds unreported mass must. B8′s histogram is the flattest of the three delay-aware models—no bin departs from 0.100 by more than 0.012, compared to 0.017 for B5 and 0.032 for B4—and its intervals are the narrowest (80% width 0.7113, against 0.9476 for B4 and 0.8766 for B5). Yet, its pooled WIS is the worst of the three (Figure 4B). Distributional calibration and predictive accuracy are not the same property here: central error is a major contributor to B8′s poorer WIS (MAE 0.3390 against 0.2589 for B5), although its positive-target undercoverage shows that its uncertainty quantification is not problem-free either.

#### 3.3.1 Accuracy and calibration of the exploratory two-part count model

Continuous quantile regression assigns no probability mass to zero and does not have count support, which is poorly suited to a target that equals zero in 86.1% of scored cells. The exploratory two-part boosted model described in Section 2.5.1 (Z1) tested whether an explicit count formulation improved calibration. Z1 had a pooled macro-WIS of 0.1939, a 9.6% improvement over B4 that would have cleared the 5% gate, although its score remained 11.0% higher than B5 using the same denominator convention. Z1 differed most clearly from B5 in calibration on positive-target cells: its 80% and 95% coverage was 78.6% and 89.9%, compared with 70.8% and 88.5% for B5, and its 80% intervals were narrower on those cells (3.00 vs. 3.79). Its positive-target WIS of 1.200 fell between B5 and B4 (Figures 4A, C). For the event that at least one outbreak-equivalent remained unreported, whose observed rate was 7.83%, Z1 achieved a Brier score of 0.0516, compared with 0.0587 for B5, 0.0614 for B4, and 0.0655 for B8. Its mean predicted probability of 9.15% was closest to the observed rate, compared with 12.65% for B5 and 14.60% for B4 (Figures 4D, E). The probability estimates were constructed differently: Z1 used a logistic booster fitted directly to the event, while B4 and B8 derived the probability from their count distributions, and B5 used the fixed transformation of its predicted mean described in Section 2.3.1. The comparison therefore describes the probability that each method supplied to a surveillance analyst; it does not isolate the effect of the two-part formulation on probability estimation. An explicit point mass at zero improved positive-target coverage and alert-probability calibration relative to B5, at the cost of approximately one-tenth of B5's WIS advantage. Z1 was fitted after the completion of the locked evaluation and did not enter the adoption gate or replace B5 as the best-scoring prespecified method.

#### 3.3.2 Contribution of zero-mass calibration to the measured advantage

The two delay-aware comparators are not misspecified in the way B5 is: both emit a proper count distribution through the Gamma–Poisson update, so both already place mass at exactly zero. Whether they place enough is a different question, and the zero-inflated variant of Section 2.5.1 answers it at a cost of one mixing weight per cohort age, fitted on validation and frozen before the locked test. Both models assigned insufficient probability to zero, and the shortfall widened with cohort age: the fitted weights rise from 0.25 at age 0 to 0.46 at age 3 for B4, and from 0.00 to 0.49 for B8. That B8 needs no excess at age 0 fits the age-dependent pattern of Section 3.4: its weakness at the newest week is an error in the central estimate, not a misplaced zero. Supplying the missing mass improved both models more than a recalibration of a well-specified model should: B4 improved by 18.8% (0.2145 to 0.1741; paired cutoff-block interval on the difference −0.0509 to −0.0308); B8 improved by 10.9% (0.2294 to 0.2044; −0.0301 to −0.0202). Positive-target coverage rose in both models, from 79.1% to 82.1% at nominal 80% for B4 and from 68.2% to 75.2% for B8, indicating that the gain was not achieved by collapsing the positive cells (Figure 4F; Table 3).

After an explicit zero mass was added to B4, its WIS was 0.1741, compared with 0.1748 for B5. Expressed as B4 with zero mass relative to B5, the difference was −0.4%; the paired absolute WIS difference was −0.0007 (95% cutoff-block interval −0.0071 to 0.0059). Because the interval included zero, this exploratory analysis did not detect a difference between the recalibrated B4 and B5. This does not provide evidence of equivalence. B5 and B4 differ in learner, feature construction, target, and predictive distribution, so the analysis cannot partition the original margin among these components. It supports only the conclusion that zero-mass calibration accounts for a substantial part of the measured gap. Under the same recalibration, B8 with zero mass scored 0.2044, compared with 0.1741 for B4 with zero mass. B8 was therefore 17.4% worse; the paired absolute WIS difference was 0.0303 (95% cutoff-block interval 0.0215 to 0.0401). Insufficient zero mass did not explain B8's deficit in this analysis. These recalibrations used stored predictions rather than refitted models and were exploratory; the prespecified comparison between B8 and B4 remained unchanged, and B5 continued to be the best-scoring method under the frozen protocol.

### 3.4 Effect and transferability of the reporting update

Removing the reporting layer isolates its contribution. Pooled over ages 0–3, B8 scored 17.0% below its matched no-delay counterpart (0.2294 vs. 0.2762 for B6; difference −0.0468, 95% cutoff-block interval −0.0610 to −0.0341), and B9 scored 18.0% below B7 (0.2284 vs. 0.2786; −0.0503, −0.0659 to −0.0359). The pooled figure conceals a reversal: at age 0, B8 was 24.7% worse than B6 (0.3646 vs. 0.2924), then 3.3% better at age 1, and 36.5%, 56.5%, and 85.4% better at ages 2, 3, and 8 (Figure 5D; Supplementary Table S6). The per-age profiles explain this crossover: the no-delay variants are nearly flat across cohort ages (0.2924 at age 0, still 0.2671 at age 8) because they never incorporate what has already been reported, whereas the raw observed curve improves roughly 15-fold over the same span (0.5263 to 0.0344); the delay-aware network sits between the two and crosses over between ages 0 and 1 (Figure 3B), so conditioning on the observed count helps once there is enough data to condition on, and hurts before that.

**Figure 5:**
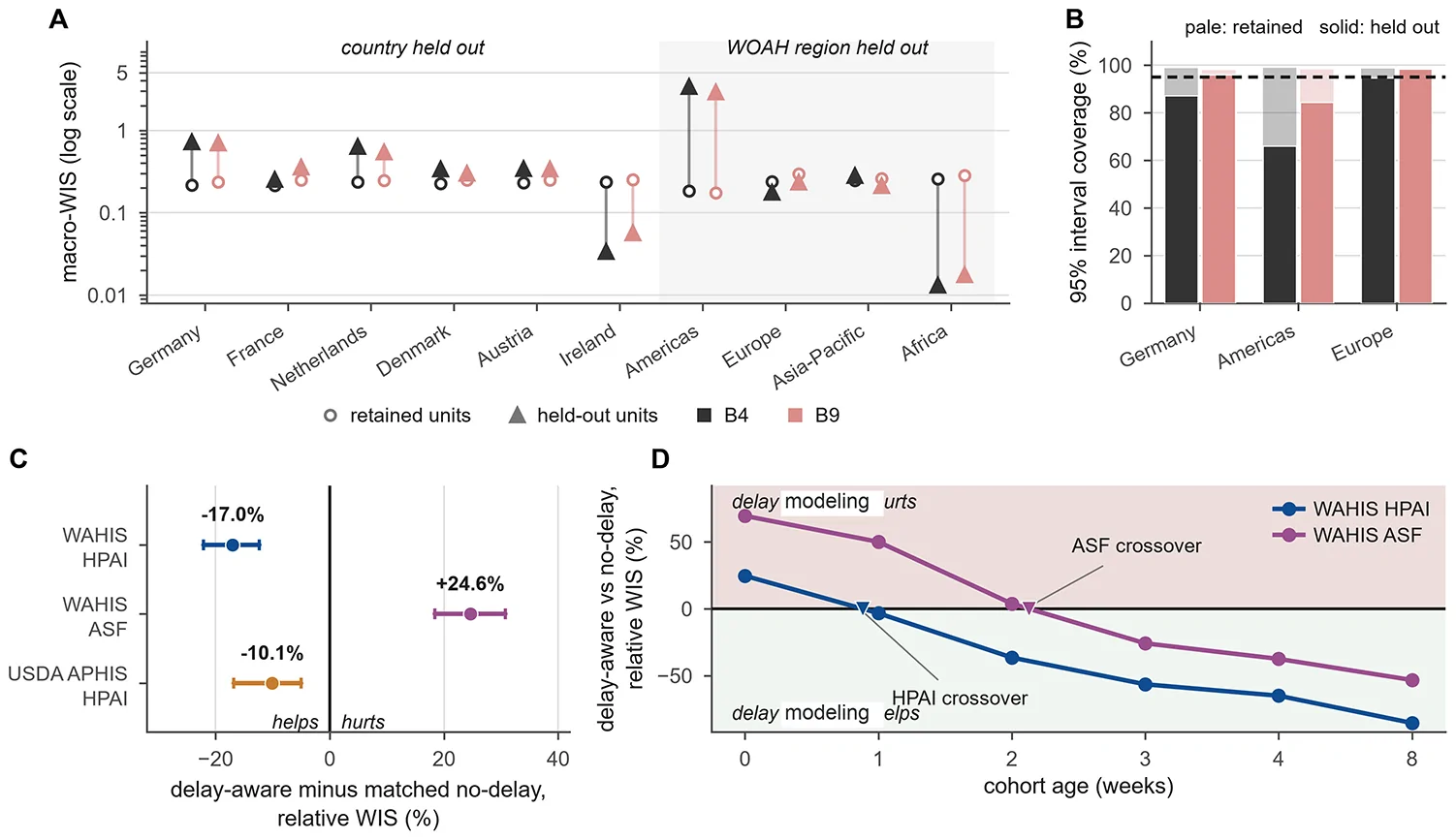
The boundaries of generalization. **(A)** Leave-one-country and leave-one-region refits: macro-WIS on the held-out units (filled triangles) vs. the units retained in training (open circles), logarithmic scale. Degradation tracks how much a held-out unit reports. **(B)** 95% interval coverage for the same refits; pale bars represent retained units, solid bars represent held-out units, and the dashed line indicates nominal. **(C)** Matched delay-aware vs. no-delay contrast in three surveillance systems with 95% paired bootstrap intervals; the APHIS comparison follows system-specific retraining. **(D)** The same contrast by cohort age in HPAI and ASF. Triangles mark the crossover point at which delay modeling begins to provide benefits; it sits between ages 0 and 1 in HPAI and between ages 2 and 3 in the slower-reporting ASF system.

The same framework did not transfer to ASF, where the completeness profile is slower. Pooled over ages 0–3, B8 scored 1.6117 against 1.2937 for B6-−24.6% worse (difference +0.3181, 95% cutoff-block interval +0.2377 to +0.3974), with 25.8% in the graph-carrying pair (+0.3341, +0.2539 to +0.4135; Table 4; Figure 5C). This inverts the HPAI result, so the prespecified cross-disease criterion of a directionally consistent delay-aware advantage is not merely unmet but reversed. The per-age profile shows the same crossover as HPAI, only displaced: B8 was 69.5% worse at age 0 and still 3.8% worse at age 2, then 25.8% and 53.3% better at ages 3 and 8 (Figure 5D). ASF completeness is 6.7% at age 0 and 31.3% at age 1, compared to 7.6% and 36.2% for HPAI, so the noise-amplifying region extends about 2 weeks further; three of the four prespecified scored ages fall inside it for ASF compared to one for HPAI, which is what inverts the pooled comparison.

**Table 4:** Matched delay-module comparisons across surveillance systems.

System	B8 WIS	B6 WIS	B8–B6 (95% CI)	Relative	Interpretation
WAHIS HPAI	0.2294	0.2762	−0.0468 (−0.0610, −0.0341)	−17.0%	Delay-aware better
WAHIS ASF	1.6117	1.2937	+0.3181 (+0.2377, +0.3974)	+24.6%	Delay-aware worse
USDA APHIS HPAI	1.1496	1.2794	−0.1298 (−0.2149, −0.0640)	−10.1%	Delay-aware better

B8 is delay-aware B6; relative = (B8–B6)/B6, with negative values favoring B8. Intervals are paired cutoff-block bootstrap. The APHIS comparison was prespecified and follows APHIS-specific retraining of model weights; the WAHIS intervals are exploratory. WAHIS and APHIS targets differ, so absolute scores are not comparable across rows.

The complexity gate applied to ASF, using that disease's own strongest transparent comparator, reproduced the HPAI verdict. B4 was not that comparator here—it scored 1.6828 and was surpassed by the NobBS-style smoother at 1.5359, which became the ASF reference. Against it, B5 improved by 38.2% (difference interval −0.7005 to −0.4733) and cleared the gate, while B8 missed it by 4.9% (0.0431 to 0.1092) and B9 by 6.0% (0.0546 to 0.1331). The ordering of approaches—gradient boosting first, followed by the deep delay-aware models behind a transparent comparator—thus holds for a second disease with a different host mix and a longer reporting tail, even though the contribution of the delay module itself reverses (Supplementary Table S3). Nor is the ASF inversion explained by the 52-week truncation or by a common pattern of completeness overestimation: bias was −1.77 for the raw observed curve, −0.82 for the NobBS-style smoother, and −0.77 for B8, but only −0.04 for the global ratio estimator and +2.93 for the country–host ratio estimator. A single truncation artifact would not produce that spread; the age-dependent pattern is more consistent with the observed reversal.

In the separately frozen APHIS transfer evaluation, B8 scored 1.1496, an improvement of 10.1% over its matched no-delay network (1.2794; absolute difference −0.1298, 95% cutoff-block interval −0.2149 to −0.0640). It was essentially tied with B4 (1.1503; relative difference 0.06%, interval on the absolute difference −0.0341 to 0.0335) and 11.1% worse than B5 (1.0347; interval 0.0514 to 0.1919). Coverage was 84.8% for 80% intervals and 90.1% for 95% intervals, both within the gates frozen on WAHIS. Because model weights were retrained on APHIS development data, this provides evidence that the reporting-delay component and the evaluation protocol carry over to a second HPAI reporting system, measured against a matched no-delay network fitted the same way. It does not validate a fixed fitted model, does not provide evidence of deep-model superiority, and does not demonstrate that APHIS wild-bird detection records agree with WAHIS outbreak counts. This APHIS interval was part of the frozen inference plan; the WAHIS delay-module intervals quoted above were computed after the test using the same procedure and are treated as exploratory.

Spatial extrapolation produced the weakest results and defines an important limit on applicability. Refitting with individual countries and entire WOAH regions held out, held-out performance tracked how much the held-out unit reports rather than how novel it is (Figure 5A). For the two heaviest reporters, the degradation was severe: with Germany held out, B4 scored 0.7165 on Germany against 0.2159 on the units it retained, and with the Americas held out it scored 3.3852 against 0.1835, with 95% coverage falling from 98.9% to 66.0% (Figure 5B). B9 behaved similarly (0.6948 and 2.9066, coverage 84.5% in the Americas). Sparse holdouts resulted in low held-out scores (0.0335 for Ireland, 0.0130 for Africa) because those units report very little, not because extrapolation succeeded there. Neither model should be used for a country or region with no reporting history in the training window.

### 3.5 Robustness, simulation, and operational value

Two design choices define the applicability of these results: the cohort rule excludes units that report infrequently, and the 52-week horizon sets the target. B5 was the best-scoring method at each of the six active-cohort thresholds examined in the exploratory analysis of Section 2.5.1 and cleared the 5% gate at all of them, indicating that the adoption decision is stable with respect to the cohort rule (Figure 6A; Table 5). Its margin widened consistently with the amount of reporting history a unit was required to have, from 15.6% on the complete 360-unit panel to 26.0% among the 38 units with at least 50 development-period outbreak equivalents (Figures 6A, B; Table 5).

**Figure 6:**
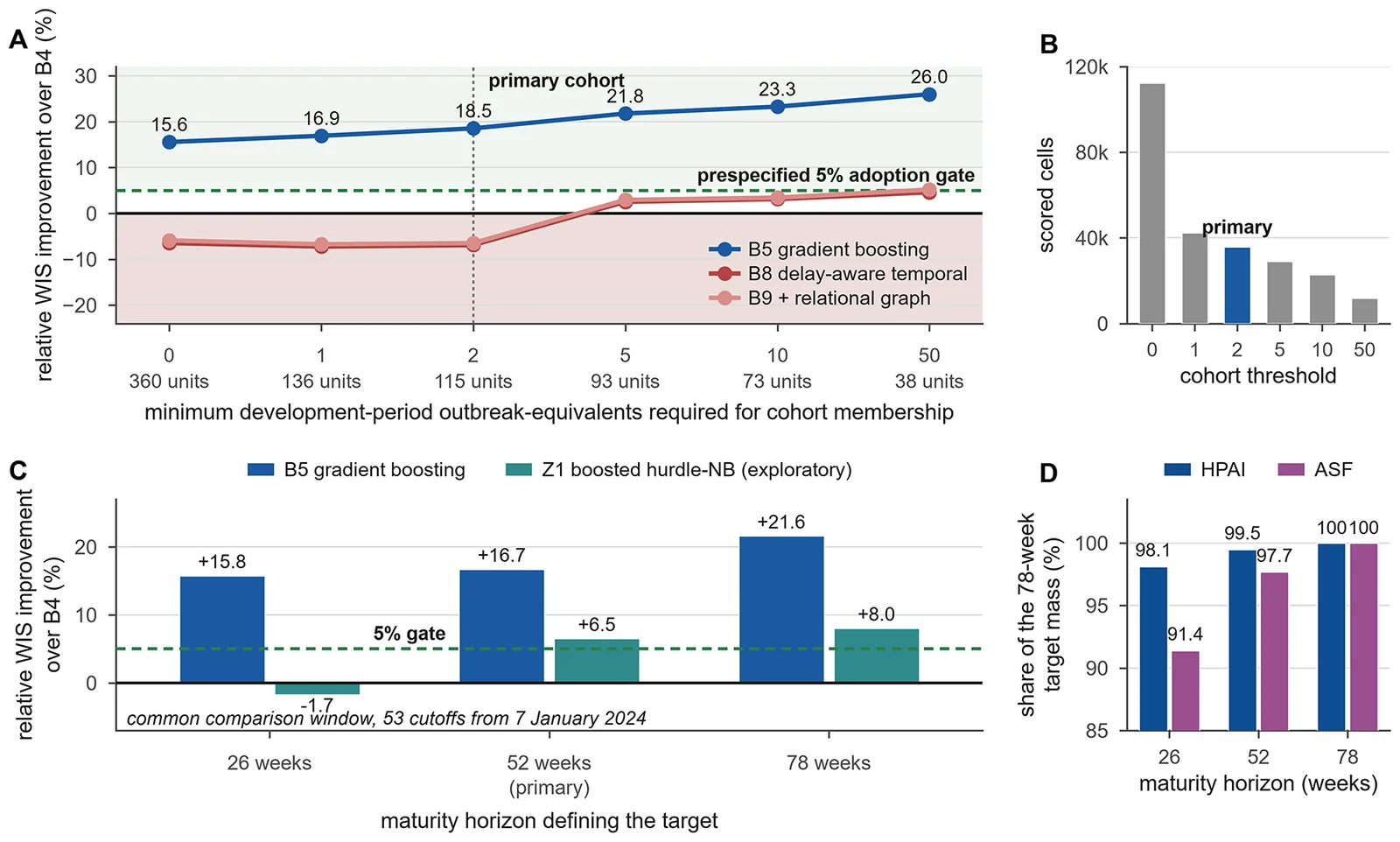
The adoption decision under alternative cohort definitions and maturity horizons. **(A)** Relative WIS improvement over B4 at six active-cohort thresholds. Gradient boosting clears the 5% gate at every threshold, and its margin widens monotonically with the reporting history a unit is required to have; the deep models clear it only in the most restrictive, exploratory definitions. **(B)** Scored cells retained at each threshold. **(C)** Relative improvement at 26-, 52-, and 78-week maturity horizons, each a rebuilt target with the compared methods refitted on a common 53-cutoff window. Only B5 maintains its position across all three. **(D)** Share of the 78-week outbreak-equivalent target mass captured at each horizon, by disease.

**Table 5:** Robustness of the adoption decision to the active-cohort rule and the maturity horizon.

Analysis	Definition	Units	Cells	B5 vs. B4	B8 vs. B4	B9 vs. B4
Active cohort	≥0 outbreak-equiv. (full panel)	360	112,320	+15.6%	−6.5%	−5.9%
	≥1	136	42,432	+16.9%	−7.2%	−6.7%
	≥2 (primary)	115	35,880	+18.5%	−6.9%	−6.5%
	≥5	93	29,016	+21.8%	+2.5%	+2.9%
	≥10	73	22,776	+23.3%	+3.1%	+3.4%
	≥50	38	11,856	+26.0%	+4.6%	+5.2%^a^
Analysis	Definition	Units	Cells	B5 vs. B4	Z1 vs. B4	Mass^b^
Maturity horizon	26 weeks	115	24,380	+15.8%	−1.7%	98.1/91.4
	52 weeks (primary)	115	24,380	+16.7%	+6.5%	99.5/97.7
	78 weeks	115	24,380	+21.6%	+8.0%	100/100

Values are relative macro-WIS improvements over B4; positive values favor the listed model. The prespecified adoption gate is +5%. Cohort rows rescore the frozen locked-test predictions at each threshold; horizon rows rebuild the reporting tensor and refit the listed methods, and are compared on the common window of 53 cutoffs from 7 January 2024 to 5 January 2025, for which age-0 targets are mature under all three horizons. Only B5 holds its position across every row; the ordering of the methods behind it changes with both the cohort rule and the horizon.

^a^The single condition under which the graph model would have passed the gate, on an exploratory cohort restricted to the 38 heaviest reporters. It is reported for completeness and is not a prespecified analysis.

^b^Percentage of the 78-week outbreak-equivalent target mass captured at that horizon, HPAI/ASF.

The cohort rule does affect the ranking of methods below the leader. At the three lowest thresholds, the transparent comparator ranked second, ahead of both neural models; from the ≥5 threshold upward, it ranked last of the four, because the neural models improved on restriction to high-volume units more rapidly than it did. Only the most restrictive definition changed the adoption decision: B8 never cleared the gate, moving from 6.5% worse than B4 on the full panel to 4.6% better among the heaviest reporters, while B9 improved further to reach +5.2% at the last threshold—the only evaluated cohort under which the primary neural hypothesis would have been supported, albeit with the narrowest applicability. This exploratory cohort discards 89% of the panel and is not a basis for changing the primary conclusion. Nor does it contradict the overall model ranking: the gate assesses whether a neural model outperforms the transparent comparator by 5%, not which method is best. In the same 38-unit cohort where B9 reaches +5.2%, B5 achieves +26.0%, and the margin between the two is greater there than anywhere else in the panel. Narrowing attention to high-volume reporters strengthens the case for the simpler method rather than undermining it.

Varying the maturity horizon over 26, 52, and 78 weeks changed the numbers while leaving the decision intact: B5 cleared the gate at all three, improving on B4 by between 15.8% and 21.6% in the common 53-cutoff window (Figure 6C). Here too, the ordering below the leader shifted: the exploratory two-part model of Section 3.3.1 surpassed B4 by 6.5% and 8.0% at 52 and 78 weeks, respectively, but lagged 1.7% behind it at 26 weeks, with only the position of B5 remaining stable across all three horizons. The 52-week choice approaches the asymptote of what a longer horizon would add: a 52-week target captures 99.5% of the outbreak-equivalent mass that a 78-week target captures in HPAI, and 97.7% in ASF; a 26-week target captures 98.1% and only 91.4% (Figure 6D). Shortening the horizon incurs substantially greater costs in the slower-reporting disease than lengthening it gains in either, which is the quantitative argument for 52 weeks. The same figures indicate the direction of the residual bias: the horizon is finite, so every estimate here is of eventual official reports within a year, and in ASF, roughly one report in forty-three arrives too late to be counted at all.

Three further lines of evidence bear on how far the adoption decision travels. Across 100 replicates in each of ten scenarios with known truth, the delay-aware estimator recovered the generator's completeness more accurately than a fixed curve in nine scenarios (mean absolute error 0.064 vs. 0.100), and every scenario exceeded 90% empirical coverage for nominal 95% intervals (Supplementary Figure S2). In the stable-delay scenario, where a single fixed curve was the true mechanism, the fixed estimator was an order of magnitude more accurate (0.004 vs. 0.038); adaptivity provides robustness to drift at some cost in efficiency when there is no drift, and the real-data hazard tests indicate which regime applies here. Under permanent deletion before reporting, with a quarter of true events removed, the estimand covered 74.9% of the true burden, and the estimator recovered the estimand rather than the burden—which quantified the identifiability boundary defined in Section 2.1. Excluding all 1,543 mammalian-host HPAI outbreaks—dominated by the dairy-cattle H5N1 notifications filed under the non-poultry label before a bovine-specific label existed (41)—left the conclusions unchanged: B5 improved 18.6% over B4, B8 was 2.6% worse than B4, the delay-module contrast represented a 13.7% improvement, and the Spearman correlation of model scores with the primary analysis was 0.993. How that was computed mattered: those outbreaks constitute 4.0% of retained HPAI outbreaks but account for 19.2% of the matured target mass in the locked-test active cohort, and the shortcut analysis—recompute the target, rescore the saved predictions, skip refitting—produced a sign reversal (+10.9%) and a rank correlation of 0.70. Models fitted to a target containing a large, bursty, geographically concentrated component are handicapped when scored against a target without it, especially when the observed count is used most aggressively; a full rebuild and refit removed the artifact entirely. That experience is why the maturity-horizon analysis above was implemented as a rebuild, and equally why the cohort-threshold analysis was not: a redefined target invalidates stored predictions, whereas a redefined scoring cohort leaves every model input exactly as it was. Cluster and target definitions did not reverse the conclusion either (delay contrast −17.0% cluster-expanded, −15.9% one-per-identifier, −15.9% clusters excluded).

In the operational analysis, the ranking signal had a larger effect than the choice among the stronger models. Under a weekly review budget of 20 country–host units, ranking by observed count recalled 24.9% of the units with at least one unreported outbreak-equivalent, at 5.61 units investigated per unit found. Ranking the same units by expected unreported count recalled 64.5%, and by the probability that at least one outbreak-equivalent remained unreported, 64.8%, corresponding to a 2.6-fold gain in recall with the number needed to investigate falling to 2.15 (Figures 7A, B). At a budget of five, the best recall rose from 12.2% to 24.4%. The observed-count ranking tied at the selection boundary in all 78 cutoffs (median tie 112 units), so its value reflects the exact expectation over tied units rather than an artifact of input order. Once the ranking signal is fixed, the stronger models cluster: 0.648 for B4, 0.639 for B5, and 0.618–0.621 for the neural models, with differences far smaller than the gap from observed-count ranking (Figure 7C). Ranking requires only a useful ordering, which is a weaker demand than the calibrated magnitudes that WIS scores. Lead time, however, is modest: flagging units whose estimated probability that at least one outbreak-equivalent remained unreported exceeded 0.5, B4 flagged 957 country-weeks and preceded the first visible official report in 64.7% of them (median lead 1 week, mean 1.45); B5 flagged 858 and preceded it in 74.6% (mean 1.59); B8 flagged 1,095 but only 36.0% (median zero), consistent with its age-0 weakness. This lead is measured against an official report becoming visible, not against laboratory confirmation or true infection.

**Figure 7:**
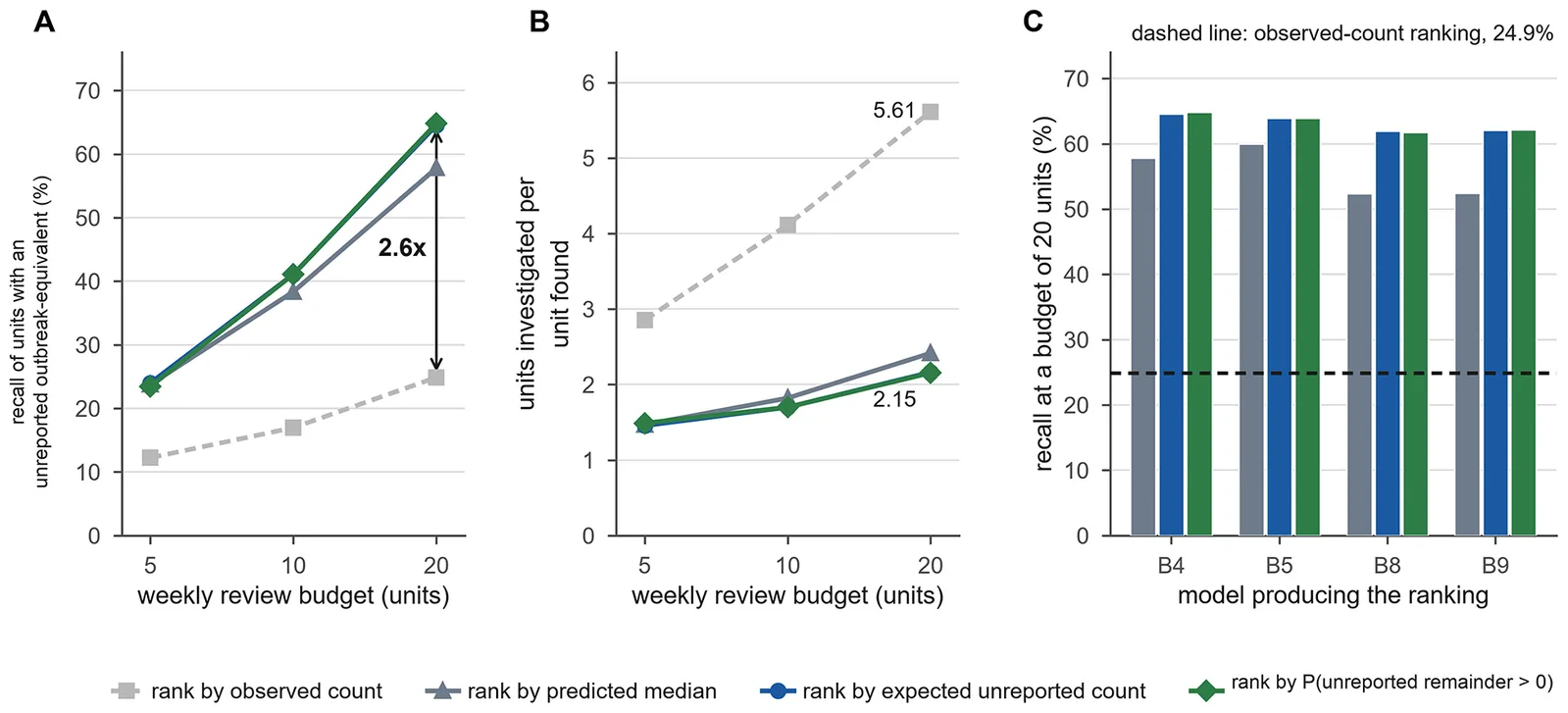
Decision value in the primary HPAI evaluation under fixed weekly review budgets. **(A)** Recall of country–host units with at least one unreported outbreak equivalent, by the signal used to rank them. **(B)** Units investigated per detected unit. **(C)** Recall at a budget of 20 units across four models and three model-based ranking signals; the dashed line represents the naive observed-count ranking. The gain comes from changing the ranking signal rather than from adopting a specific model.

## 4 Discussion

### 4.1 Principal findings

The prespecified primary hypothesis was not supported in the locked test. A delay-aware spatiotemporal neural model, selected on validation and frozen before test scoring, did not outperform the transparent hierarchical Bayesian nowcast, and its relational graph extension produced no material gain. The gradient-boosting comparator was the only prespecified method to clear the same gate (Section 3.2). Under the prespecified fallback rule, the transparent model therefore remains the reference, and the principal contribution is the historical-cutoff benchmark and reporting-delay analysis rather than evidence for added neural complexity. The gradient-boosting advantage was smaller in scope than the pooled estimate suggested. It depended on reporting volume: gradient boosting outperformed B4 by 26.0% among the 38 highest-volume country–host units but performed worse among units with almost no reporting history. The analysis could not partition the margin among learner, feature, target, and distribution effects. The locked test therefore supports a specific conclusion: one gradient-boosting formulation was the best of twelve prespecified methods for units with an established reporting history. It does not establish that gradient boosting is preferable for animal-disease nowcasting in general or that deep spatiotemporal architectures are generally inferior.

The exploratory zero-mass analysis identified a plausible contributor to B5's advantage without isolating a single mechanism. B5 and B4 differ in learner, feature construction, target formulation, and predictive distribution. Adding an explicit zero mass to B4 removed most of the measured gap, which is consistent with zero-mass calibration contributing substantially, but the other differences were not held fixed (Section 3.3.2). The same recalibration did not eliminate B8's deficit: B8 remained 17.4% worse than B4 under identical treatment. B8's poorer WIS had a substantial central-accuracy component despite its relatively flat probability integral transform and narrow intervals (Section 3.3). Estimation error may accumulate because B8 estimates incidence and reporting hazard separately before combining them, using 115 units in a panel where 86.1% of scored cells have a zero mature target. The design cannot determine whether a different architecture, a longer training window, or a larger panel would reduce this gap. It establishes only that the architecture used for this evaluation did not do so.

Several findings did not depend on which model performed best. At week closure, 7.6% of eventual HPAI and 6.7% of eventual ASF outbreak equivalents were visible. A decline at the end of an official animal-disease series can therefore be dominated by reporting delay. A fixed delay distribution was misspecified for these data, and the known-truth simulation reproduced the benefit of adaptation except when a fixed curve was the data-generating mechanism. These findings agree with human-disease nowcasting studies in which reporting distributions varied over time and place (14, 15, 42). The present analysis quantifies that variation in a multicountry official animal-health system and shows that it persists in out-of-time evaluation. Host structure also mattered: domestic and wild reporting pathways differed by approximately a factor of three in the ASF delay tail.

### 4.2 Age-dependent reversal of the reporting update

The reversal follows from the low completeness of the newest cohort. At age 0, the implied inflation between the visible count and the eventual total is about 13-fold, so a small absolute error in estimated completeness becomes a large error in the implied total. Delay correction is thus most valuable exactly where it is hardest, and the model that ignores the partial count and predicts from matured history alone is better off until enough of the count has arrived to be worth conditioning on. The crossover occurs between ages 0 and 1 in HPAI and between ages 2 and 3 in ASF, and its position is determined by the completeness curve of the reporting system rather than by the pathogen. The ASF result indicates that transfer depends on the completeness profile of the reporting system rather than on disease identity alone.

The graph result applies only to the relationships and spatial scale evaluated here. Geography, live-animal trade, and cross-host coupling, as encoded here, carried no information that the reporting history of a unit did not already convey, but the encoding is at the country level, its trade edges are a development-period average, and 20 of 180 countries had no polygon match, thus retaining only trade and cross-host edges. A finer spatial unit, a time-varying trade network, or a formulation that propagates completeness rather than counts could behave differently. Edges here represent predictive relationships: an attention weight is not a transmission pathway, and a country embedding carries no political meaning.

### 4.3 Scope of inference

Interpretation of these results is bounded by the estimand and the study cohort. Every number reported here pertains to the 52-week official-report target defined in Section 2.1 and does not estimate incidence, prevalence, or true burden; the deletion scenario quantifies this gap (Section 3.5). The primary evaluation covers 115 country–host units that recorded at least two outbreak equivalents during development. Units that report infrequently were excluded by this rule, and the threshold analysis shows that including them reduces, rather than reverses, the advantage of gradient boosting (15.6% on the full panel vs. 18.5% in the primary cohort). Because the cohort is not a random sample of WAHIS members, the results should not be applied to a member state without an established reporting history.

The scope of inference is also limited by the architectures and the external systems evaluated. Twelve methods were compared, including one causal transformer and one relation-aware graph block. The evidence, therefore, applies to these architectures at the tested capacities on this panel and does not support a general claim about deep learning for veterinary surveillance. The graph model crossed the adoption threshold only when scoring was restricted to the 38 heaviest reporters, an exploratory condition reported in Section 3.5. The APHIS analysis retrained model weights and therefore evaluates the transfer of the architecture and protocol after system-specific retraining, rather than validating a fixed fitted model. It does not establish broad generalizability across surveillance systems because its target comprises wild-bird detection records in one country and for one disease, which are not numerically interchangeable with WAHIS outbreak counts.

Finally, the evaluation was a retrospective replay of a frozen snapshot rather than a prospective deployment. Every model was fitted to historical delay behavior and scored against outcomes that had already matured within the same snapshot. This design cannot establish how a model performs when the reporting process changes; the changepoint scenario constructed to represent such a change was the worst calibrated of the ten. Nor can a retrospective replay capture the feedback created by deployment, where investigating a flagged unit may lead to earlier reporting. The available extraction cannot resolve this limitation because mature outcomes require 52 weeks of follow-up, the final locked cutoff occurs 52.6 weeks before the snapshot ends, and the locked evaluation uses the entire mature period available, leaving no later interval for validation. Prospective evaluation therefore requires a new WAHIS extraction with predictions registered before reports arrive. Until such an evaluation is conducted, the current evidence should not be used alone to support claims about operational performance in a live system.

### 4.4 Implications for surveillance practice

The observed count alone was a poor prioritization signal in this retrospective evaluation: under an unchanged weekly budget, ordering units by the estimated probability that at least one outbreak-equivalent remained unreported raised recall and yield by about 2.6-fold. The stronger models produced similar orderings despite differing scores, suggesting that the ranking signal may matter more than model class, although this potential operational benefit requires prospective confirmation. A flagged unit has a higher estimated probability of an unreported outbreak-equivalent, but this does not provide evidence that a country failed to report or that an unobserved outbreak occurred; if the ranking signal is what matters, the probability behind it should be the calibrated quantity, on which the two-part count model of Section 3.3.1 performed best.

Zero-mass calibration should be examined before model classes are compared on a sparse count panel. Here, one weight per cohort age, fitted on historical validation data and applied to a Gamma–Poisson nowcast, improved WIS by 18.8% and reduced the measured distance to the boosted model below what this exploratory comparison could resolve. This preserves the structure and interpretability of the hierarchical nowcast, but its value outside this panel requires independent validation.

The delay-correction window should be derived from the completeness curve of the target system before the correction is activated, rather than inherited from the system where the method was developed. Operationally, that means estimating completeness by cohort age on local historical data, identifying the age at which the corrected estimate begins to outperform one that ignores the partial count, and applying the correction only from that age onward. In our HPAI panel, that crossover occurred between ages 0 and 1; in the slower-reporting ASF panel, it occurred between ages 2 and 3, and applying the HPAI window there would have made the nowcast worse than doing nothing.

The observed curve and the expected mature official-report curve should be drawn as separate series on any dashboard, with completeness by cohort age displayed alongside them, allowing users to see how much of the newest week is missing rather than inferring a downturn from it. For that newest week, the display should either down-weight the partial-count update or revert to the prior-dominated estimate, and should never provide a point estimate without an interval; in cases where a unit reports infrequently, it should revert to the transparent estimator, which was the better model in that stratum. None of these recommendations is converted into a cost–benefit figure because credible investigation costs and under-reporting losses were unavailable.

### 4.5 Strengths and limitations

The strengths of the design include a fixed source extraction, exact reconstruction of report-date visibility, a development-defined cohort, a single locked out-of-time test with all methods rerun together, matched delay and graph ablations, multi-level clustered uncertainty, a known-ground-truth simulation, a separately frozen second-system evaluation with retraining, and the reporting of negative results and protocol deviations. Reporting positive-target coverage exposed calibration weaknesses that the zero-dominated overall summary would have obscured. The additional analyses utilized implementations appropriate to their questions: Z1 was newly fitted, the zero-mass analysis recalibrated frozen predictive distributions, the cohort-threshold analysis re-aggregated frozen predictions, and the maturity-horizon analysis rebuilt the target and refitted the compared methods. All were excluded from the prespecified adoption decision.

Several limitations bound the claims, beyond the boundaries set out in Section 4.3. Measured delay reflects detection capacity, laboratory confirmation, administrative processes, wildlife sampling intensity, and notification rules jointly; it cannot be decomposed into any one of them and must not be interpreted as a national transparency score. Report dates support the reconstruction of historical visibility, but the extraction contains only the final recorded onset date and no version history. Therefore, onset-date revisions cannot be replayed. The final recorded onset date preceded the first report for 99.8% of outbreaks. The snapshot also carries survivorship, as records deleted before extraction cannot be audited from it. The pooled ages 0–3 window was fixed before the test, and the ASF inversion shows that the window itself, rather than the method, can determine the result: a study scoring a different age range would reach a different pooled conclusion from the same predictions. Sparse structural zeros inflate aggregate coverage, which is why positive-target and volume-stratum analyses are reported rather than a single pooled coverage figure. Disease and host definitions followed exact official labels, but a WAHIS English disease name maps to as many as 22 disease identifiers, so selection operates at the name level; independent veterinary review of that label mapping is not part of the record accompanying this study. What bounds its influence is the published identifier inventory and the full mammalian-exclusion refit, which showed that the conclusions do not depend on the one label boundary that carries material mass. The planned global ERA5-Land climate feature suite was unavailable; an exploratory regional archive covering nine active-cohort countries provided an apparent 0.74% improvement, with both cutoff- and country-block intervals crossing zero, and a 1.94% deterioration within covered units, which does not support a stable climate gain (43, 44). The 5% gate was a study-defined adoption threshold, not a literature-derived biological one.

## 5 Conclusion

Heterogeneous reporting delay distorts the most recent weeks of official animal-disease curves severely enough that they should not be interpreted without correction. Correcting for it helps, but over a narrower window than expected: the correction was less effective than ignoring the observed count at the newest week and increasingly valuable thereafter. In the more slowly reporting ASF system, that harmful region covered three of the four scored ages and reversed the pooled comparison. The window should be chosen from the completeness curve of the target system rather than transferred from the system where the method was developed. The tested deep spatiotemporal architecture did not improve accuracy over the transparent comparator, and the relational graph produced no robust gain at matched capacity. After APHIS-specific retraining, the results support the transfer of the delay-aware architecture and evaluation protocol against a matched no-delay network, not deep-model superiority or cross-system count equivalence.

Among the twelve prespecified methods, gradient-boosted quantile regression provided the best probabilistic accuracy under the studied surveillance conditions: a retrospective replay of one frozen WAHIS extraction, restricted to country–host units with an established reporting history, scored over cohort ages 0–3 against a 52-week official-report target. However, the advantage is qualified. It is concentrated in the units that report the most, it comes with under-coverage where the target is positive, and zero-mass calibration appears to explain a substantial part of the measured gap: giving the transparent nowcast an explicit mass at zero improved it by 18.8%, after which no difference between the two was detectable in this exploratory comparison. Since the models differ in more than just their predictive distribution, this does not isolate a learner effect or establish equivalence. The same correction did not close the deep model's gap, so insufficient zero mass does not explain the failure of added neural complexity.

Performance deteriorated sharply when high-volume countries or regions were held out; therefore, these results should not be extrapolated to a member state without an established reporting history. Under an unchanged review budget, ranking units by the estimated probability that at least one outbreak-equivalent remains unreported rather than by the observed count raised recall and yield by about 2.6-fold. The framework serves situational awareness and the prioritization of future official reports; it does not reconstruct unobserved infections, judge national reporting transparency, or substitute for a prospective evaluation.

## Data Availability

The source data analyzed in this study were obtained from the publicly accessible WOAH World Animal Health Information System (1) and the public USDA APHIS source cited in the article. Applicable source-provider terms govern redistribution of the exact downloads. The published Supplementary Material contains the additional methods, audit details, and aggregate results. The frozen analysis code, protocols, manifests, checksums, and locked per-row prediction files used to reproduce the reported HPAI, ASF, and APHIS WIS tables will be made available by the authors, without undue reservation, upon reasonable request. Requests should be directed to the corresponding author, Xiangbo Zhu (zxb@szpu.edu.cn).
