# Supplementary material for: Nowcasting officially reported highly pathogenic avian influenza outbreak counts under heterogeneous reporting delays
Source: Front Vet Sci. 2026 Oct 8;13:1958869. doi: 10.3389/fvets.2026.1958869 (PMC13686925; doi:10.3389/fvets.2026.1958869)
Supplement: Supplementary file 1 [file Data_Sheet_1.pdf]

Supplementary Material

Nowcasting officially reported HPAI outbreaks in WAHIS: added spatiotemporal complexity did not pay, and the gradient-boosting advantage is concentrated among high-volume reporters

Kun Xiang, Xiangbo Zhu

Frontiers in Veterinary Science, Original Research

Every tabular in this file is generated directly from the frozen result artefacts by `src/build_supplementary.py`, which accompanies the submission, so no value here is transcribed by hand. Tables S9 to S12 report exploratory calibration and robustness analyses conducted after completion of the locked evaluation; none entered the prespecified adoption gate.

S1 Frozen hyperparameters, seeds and training settings
S2 Temporal encoder and relational graph, decided on validation
S3–S5 Locked-test results for every method: ASF, HPAI, USDA APHIS
S6 Macro-WIS by cohort age in all three surveillance systems
S7 Performance by development-period reporting volume
S8 Leave-one-country and leave-one-region refits
S9 Zero inflation and the two-part count model (exploratory)
S10 Zero-inflated recalibration of B4 and B8 (exploratory)
S11 Active-cohort threshold sensitivity (exploratory)
S12 Maturity-horizon sensitivity (exploratory)
S13 As-of completeness boundary convention
S14 Ten simulation scenarios with known truth
S15 Nested discrete-time reporting-hazard models
S16 Cluster handling and target definition
S17 Lead time relative to the first visible official report
S18 Hyperparameter search on validation data
Figure S1 WAHIS exclusion cascade for HPAI and ASF
Figure S2 Simulation results

1 Data processing and audit

Figure S1 traces every outbreak entity from the eligible disease labels to the reporting triangle that the evaluation scores, separately for each disease. Counts are outbreak entities rather than long-format rows, and cluster expansion is applied after this cascade, so the entity totals here and the outbreak-equivalent totals quoted in the main text are not the same quantity. Every count is taken from the frozen preflight audit, and the conservation check requires the reconstructed triangle totals to equal the eligible outbreak-equivalent totals exactly.

2 Frozen model configuration

Hyperparameters were selected on validation data with a calibration penalty and then frozen, together with the protocol, the code, the data checksum and the active cohort, before any locked-test score was computed. Nothing in Table S1 was revisited after the test opened. The full search is in Table S18.

3 Temporal encoder and relational graph

Two architecture decisions were settled on validation data with three seeds before the protocol was re-frozen, and both are reported here because one of them is a negative result that the main text relies on.

The rule applied was that a gain counts as stable only if it is positive on the mean, the seed distributions are disjoint, and the difference exceeds the pooled seed standard deviation. Under that rule the causal transformer (Vaswani et al., 2017) beat the temporal convolutional fallback (Bai et al., 2018) in the no-graph configuration (relative gain 1.11%, disjoint seed ranges) but not in the graph-carrying configuration (0.18%, overlapping ranges), so the transformer was retained. The relation-aware graph block (Schlichtkrull et al., 2018) improved WIS in only one of the six encoder-by-seed configurations, and the best no-graph configuration (0.4540) beat the best graph configuration (0.4593). The graph was therefore removed from the primary model and retained solely as the matched B9 ablation. The temporal convolutional network is about four times faster to fit; that is a convenience, it is not evidence, and it did not enter the decision.

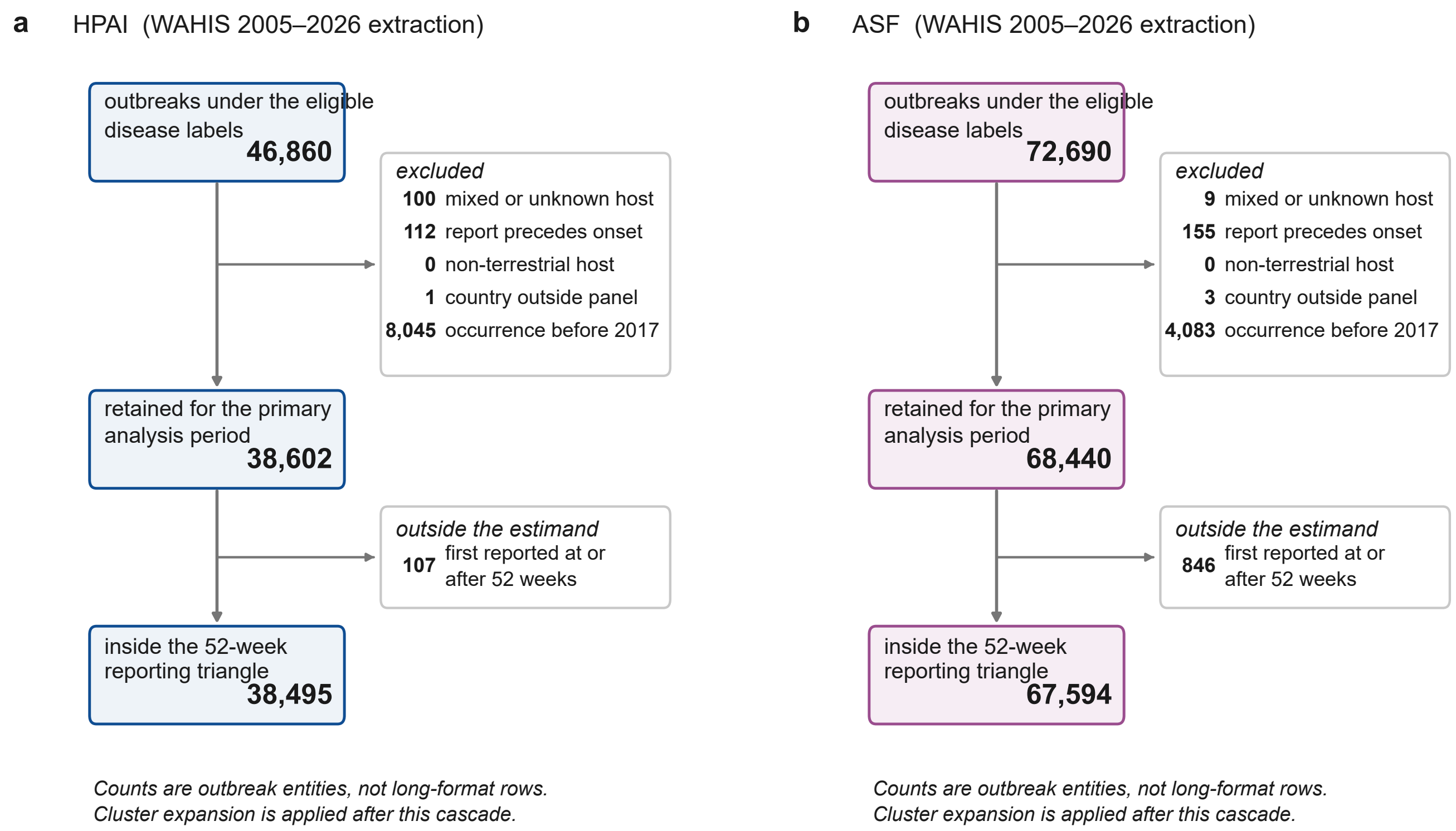

Figure S1. WAHIS exclusion cascade for HPAI and ASF. Counts are unique outbreak entities. The upper arm lists the eligibility exclusions applied before the primary analysis period; the lower arm lists outbreaks first reported at or after 52 weeks, which fall outside the estimand by construction and are retained in the audit files.

4 Locked-test results for every method

Tables S3 to S5 give all prespecified methods in each surveillance system, pooled over cohort ages 0–3. Only selected rows appear in the main text. Absolute scores are not comparable across systems because the targets differ: WAHIS counts outbreak-equivalents and APHIS counts wild-bird detection records.

Table S1. Frozen hyperparameters, seeds and training settings.

Component	Setting	Frozen value
Neural encoder (B6–B9)	temporal kind	transformer
	model dimension d	32
	temporal layers	2
	dropout	0.2
	learning rate	0.001
	graph block (B7, B9 only)	1 relation-aware layer; geography, trade, cross-host
	reporting layer (B8, B9)	discrete-time hazard, age × region × host
	predictive combination	Gamma–Poisson update
	seeds, HPAI and APHIS	11, 23, 37, 53, 71
	seeds, ASF	11, 23, 37
	ensembling	deep ensemble; within- and between-member variance
Gradient boosting (B5)	boosters	7 quantile + 1 Poisson mean
	quantile objective	`reg:quantileerror`
	trees / max depth	400 / 5
	learning rate	0.05
	row / column subsample	0.8 / 0.8
	min child weight	5
	explicit L_1, L_2	library defaults
	seed	11
	predictors	29 (Section 2.3.1)
Hurdle-NB (Z1, exploratory)	zero part	`binary:logistic`
	positive part	`count:poisson` on $U - 1$
	dispersion	NB2, method of moments on positive rows
	features, tree settings	matched to B5
Hierarchical Bayes (B4)	incidence	Poisson GLM on matured cohorts
	country ridge penalty λ	1
	own country effect requires	≥5 matured outbreak-equivalents
All methods	refit schedule	quarterly, expanding window
	quantile grid	0.025, 0.1, 0.25, 0.5, 0.75, 0.9, 0.975
	runtime	Python 3.11, pinned lock file

Table S2. Temporal encoder and relational graph on validation data, three seeds. Lower WIS is better. “Rel. gain” is the transformer relative to the TCN within the same graph setting; “Disjoint” indicates non-overlapping seed ranges; “Stable” applies the full three-part rule.

Graph	Encoder	Mean WIS	Seed range	Rel. gain	Disjoint	Stable
with graph	transformer	0.4593	0.4547–0.4621	+0.18	no	no
	TCN	0.4601	0.4578–0.4635	—		
no graph	transformer	0.4540	0.4533–0.4543	+1.11	yes	yes
	TCN	0.4591	0.4571–0.4622	—		

Table S3. WAHIS ASF locked test, all twelve methods, 54 active country–host units and 78 cutoffs (n = 16,848). The gate reference for ASF is B3, not B4.

Method	WIS	MAE	Cov50	Cov80	Cov95	Bias
B0 raw observed	1.7665	1.7665	88.2	88.2	88.2	−1.7665
B1 ratio, global	1.7894	2.1800	87.9	92.1	94.8	−0.0375
B1 ratio, region–host	1.7633	2.1436	88.5	92.2	94.8	−0.1007
B1 ratio, country–host	2.6993	3.8248	89.4	92.4	94.5	2.9288
B2 chain ladder	1.7448	2.0524	88.5	92.3	94.5	−0.2351
B3 NobBS-style	1.5359	1.9193	88.1	91.2	93.5	−0.8234
B4 hierarchical Bayes	1.6828	2.1435	88.1	91.3	93.6	−0.3378
B5 gradient boosting	0.9490	1.4591	79.2	88.8	94.6	−0.4986
B6 temporal, no delay	1.2937	2.1364	91.7	96.6	98.0	−0.5003
B7 temporal+graph, no delay	1.2943	2.0758	92.3	96.4	98.0	−0.4295
B8 delay-aware temporal	1.6117	1.9719	87.4	91.4	93.7	−0.7704
B9 delay-aware temporal+graph	1.6284	1.9844	87.4	91.2	93.6	−0.7626

Table S4. WAHIS HPAI locked test, all twelve methods, 115 active country–host units and 78 cutoffs (n = 35,880).

Method	WIS	MAE	Cov50	Cov80	Cov95	Bias
B0 raw observed	0.2894	0.2894	92.2	92.2	92.2	−0.2894
B1 ratio, global	0.2990	0.3954	92.6	94.9	96.3	0.1450
B1 ratio, region–host	0.2806	0.3591	92.9	95.2	96.5	0.1137
B1 ratio, country–host	0.3014	0.3985	92.6	94.9	96.4	0.1241
B2 chain ladder	0.2983	0.4006	92.5	94.9	97.3	0.1965
B3 NobBS-style	0.2494	0.3352	92.7	94.7	96.8	0.0014
B4 hierarchical Bayes	0.2145	0.3296	94.1	97.1	98.6	0.1782
B5 gradient boosting	0.1748	0.2589	88.2	93.4	97.5	0.0275
B6 temporal, no delay	0.2762	0.4118	95.2	97.9	99.2	0.0943
B7 temporal+graph, no delay	0.2786	0.4166	95.2	97.9	99.2	0.0815
B8 delay-aware temporal	0.2294	0.3390	92.5	95.6	98.0	0.0556
B9 delay-aware temporal+graph	0.2284	0.3363	92.6	95.7	98.0	0.0516

Table S5. USDA APHIS locked test after system-specific retraining, all ten methods, 50 active states and 31 cutoffs (n = 6,200). The relational graph was not transferred, so B7 and B9 do not appear.

Method	WIS	MAE	Cov50	Cov80	Cov95	Bias
B0 raw observed	1.3389	1.3389	74.0	74.0	74.0	−1.3389
B1 ratio, global	1.1609	1.5550	81.1	89.0	91.8	−0.2651
B1 ratio, region–host	1.1610	1.5360	81.0	88.8	91.7	−0.3032
B1 ratio, country–host	1.1879	1.5769	81.0	88.1	92.1	−0.1387
B2 chain ladder	1.1581	1.5363	80.7	89.1	91.9	−0.2587
B3 NobBS-style	1.1737	1.3785	75.7	80.0	85.4	−0.9811
B4 hierarchical Bayes	1.1503	1.3745	76.3	82.0	88.4	−0.9375
B5 gradient boosting	1.0347	1.3344	63.0	75.9	89.6	−1.0202
B6 temporal, no delay	1.2794	1.6501	81.0	87.7	92.7	−0.9188
B8 delay-aware temporal	1.1496	1.4582	78.8	84.8	90.1	−0.6226

5 Performance by cohort age and by reporting volume

Table S6 carries the per-age profiles the main text draws on. Three features matter. The no-delay ablations are nearly flat across cohort age because they never incorporate what has already been reported. The raw observed curve improves roughly fifteenfold between ages 0 and 8 as reports arrive. The delay-aware models sit between the two and cross over between ages 0 and 1 in HPAI but only between ages 2 and 3 in the slower-reporting ASF system, which is what inverts the pooled ASF comparison.

Table S6. Macro-WIS by cohort age in all three surveillance systems. Cohort ages 0–3 are the prespecified scored window; ages 4 and 8 are shown for context.

System	Method	age 0	age 1	age 2	age 3	age 4	age 8
WAHIS HPAI	B0 raw observed	0.5263	0.3262	0.1872	0.1177	0.0806	0.0344
	B4 hierarchical Bayes	0.3207	0.2646	0.1606	0.1122	0.1080	0.0546
	B5 gradient boosting	0.2696	0.1967	0.1345	0.0983	0.0789	0.0514
	B6 temporal, no delay	0.2924	0.2745	0.2673	0.2706	0.2612	0.2671
	B8 delay-aware temporal	0.3646	0.2653	0.1698	0.1177	0.0914	0.0389
	B9 delay-aware temporal+graph	0.3660	0.2623	0.1688	0.1165	0.0898	0.0383
WAHIS ASF	B0 raw observed	2.7039	2.1633	1.3635	0.8352	0.6840	0.5950
	B3 NobBS-style	1.9756	1.8600	1.3850	0.9231	0.8308	0.6735
	B5 gradient boosting	1.2750	0.9726	0.8786	0.6699	0.6294	0.6083
	B6 temporal, no delay	1.3096	1.2908	1.2838	1.2905	1.2745	1.2696
	B8 delay-aware temporal	2.2197	1.9373	1.3330	0.9570	0.7968	0.5927
USDA APHIS	B0 raw observed	1.4594	1.4174	1.2935	1.1852	1.1619	1.1555
	B4 hierarchical Bayes	1.1704	1.1781	1.1866	1.0659	1.0951	1.0644
	B5 gradient boosting	1.1180	1.0815	1.0170	0.9222	0.9039	0.9385
	B6 temporal, no delay	1.1322	1.1443	1.3235	1.5178	1.6850	1.9594
	B8 delay-aware temporal	1.1592	1.1515	1.1758	1.1118	1.1258	1.0606

Table S7 uses the full 360-unit panel rather than the balanced active cohort, because the volume strata are defined on development-period reporting and most units fall outside the active cohort. It is the analysis that shows the gradient-boosting advantage to be concentrated among units that report.

Table S7. Micro-WIS by development-period reporting volume, full 360-unit panel. Strata are 0–1, 2–8, 9–49 and ≥50 development-period outbreak-equivalents. "vs B4" is the relative improvement over the transparent comparator; positive favours the listed method.

	zero / near zero		low		medium		high	
**Method**	WIS	vs B4	WIS	vs B4	WIS	vs B4	WIS	vs B4
B0 raw observed	0.0162	−18.1	0.3373	−98.6	0.0585	−13.9	0.4711	−11.2
B1 ratio, global	0.0270	−97.3	0.2613	−53.9	0.0806	−57.1	0.5559	−31.3
B1 ratio, region–host	0.0284	−107.3	0.2660	−56.6	0.0787	−53.3	0.4976	−17.5
B1 ratio, country–host	0.0300	−119.5	0.2676	−57.5	0.0753	−46.8	0.5621	−32.7
B2 chain ladder	0.0298	−117.7	0.2594	−52.7	0.0828	−61.4	0.5537	−30.7
B3 NobBS-style	0.0148	−8.0	0.2395	−41.0	0.0601	−17.1	0.4488	−6.0
B4 hierarchical Bayes	0.0137	—	0.1698	—	0.0513	—	0.4236	—
B5 gradient boosting	0.0145	−6.3	0.1603	+5.6	0.0508	+0.9	0.3135	+26.0
B6 temporal, no delay	0.0201	−47.2	0.2664	−56.8	0.0697	−35.9	0.4927	−16.3
B7 temporal+graph, no delay	0.0188	−37.1	0.2733	−60.9	0.0720	−40.2	0.4908	−15.9
B8 delay-aware temporal	0.0141	−3.2	0.2279	−34.2	0.0560	−9.2	0.4041	+4.6
B9 delay-aware temporal+graph	0.0139	−1.6	0.2266	−33.4	0.0570	−11.0	0.4016	+5.2
cells	76,440		12,168		11,856		11,856	

6 Spatial extrapolation

Each row of Table S8 is a separate refit with the named country or WOAH region removed from training, scored separately on the held-out and the retained units. Held-out performance tracks how much a held-out unit reports rather than how novel it is: the low held-out scores for Ireland and Africa reflect units that report very little, not successful extrapolation. Neither model should be used for a country or region with no reporting history in the training window.

Table S8. Leave-one-country and leave-one-region refits. "Held-out" and "retained" are macro-WIS on the two cohorts; Cov95 is 95% interval coverage on the held-out units, against a nominal 95%.

		B4 hierarchical Bayes			B9 delay-aware temporal+graph		
**Held out**	**Scope**	held-out	retained	Cov95	held-out	retained	Cov95
Germany	country	0.7165	0.2159	87.2	0.6948	0.2347	96.0
France	country	0.2507	0.2150	94.8	0.3575	0.2488	99.4
Netherlands	country	0.6312	0.2358	87.8	0.5426	0.2467	95.4
Denmark	country	0.3364	0.2264	91.3	0.2979	0.2480	98.1
Austria	country	0.3402	0.2298	93.0	0.3359	0.2497	97.3
Ireland	country	0.0335	0.2360	98.3	0.0562	0.2515	100.0
Americas	region	3.3852	0.1835	66.0	2.9066	0.1749	84.5
Europe	region	0.1752	0.2390	94.8	0.2322	0.2955	98.4
Asia-Pacific	region	0.2795	0.2453	89.9	0.2115	0.2602	96.9
Africa	region	0.0130	0.2585	98.9	0.0176	0.2846	99.5

7 Additional calibration and robustness analyses

The four analyses in this section were specified after completion of the locked evaluation and are treated as exploratory. None entered the prespecified adoption decision or altered the frozen predictions of B0–B9. They address different questions and therefore re-run different parts of the pipeline: Table S9 reports a newly fitted model, Table S10 a recalibration of frozen predictive distributions, Table S11 a re-aggregation of frozen predictions under alternative scoring cohorts, and Table S12 a rebuilt target with refitted methods. The distinction is important because it determines what each analysis can support.

7.1 Zero inflation and the two-part count model

Continuous quantile regression places no mass at zero and has no count support, which is an awkward description of a target that is exactly zero in 86.1% of scored cells. The two-part boosted model Z1, a hurdle formulation in the sense of Mullahy (1986), tests how much of the calibration cost that mis-specification carries. Table S9 shows the result to be a trade-off rather than a clean improvement: Z1 recovers most of the positive-target coverage that B5 loses and gives the best-calibrated probability that any report remains outstanding, but it does not match B5 on WIS.

7.2 Whether the delay-aware models place enough mass at zero

B4 and B8 emit a proper count distribution through the Gamma–Poisson update, so they cannot be mis-specified in the way continuous quantile regression is, but they may still place too little mass at exactly zero. Table S10 tests that. The mixing weights were estimated on validation data by matching the observed share of zero remainders, then frozen and applied unchanged to the locked test, so this is a recalibration of stored predictions rather than a refit and no locked-test outcome entered the estimation.

Both models are short of zero mass, and increasingly so with cohort age. That B8 needs none at age 0 is consistent with its age-0 weakness being an error in the central estimate rather than a misplaced zero. Supplying the missing mass improves both and narrows the measured gap between the transparent comparator and gradient boosting to a difference whose interval contains zero — an absence of detectable difference on an exploratory contrast, not a demonstration of equivalence — while leaving the neural model 17.4% behind the transparent one under identical treatment.

Table S9. Zero inflation and the exploratory two-part count model. Scores are observation-level. "Positive mature target" restricts to the 4,981 cells whose eventual total exceeds zero; "positive unreported remainder" further restricts to the 2,810 cells where reports were still outstanding at the cutoff.

Scope	Method	n	WIS	MAE	Cov80	Cov95
all cells	B4 hierarchical Bayes	35,880	0.2145	0.3296	97.1	98.6
	B5 gradient boosting	35,880	0.1748	0.2589	93.4	97.5
	B8 delay-aware temporal	35,880	0.2294	0.3390	95.6	98.0
	Z1 boosted hurdle-NB (exploratory)	35,880	0.1939	0.2728	97.0	98.6
positive mature target	B4 hierarchical Bayes	4,981	1.3531	2.2172	79.1	90.2
	B5 gradient boosting	4,981	1.0169	1.6407	70.8	88.5
	B8 delay-aware temporal	4,981	1.5449	2.4099	68.2	85.3
	Z1 boosted hurdle-NB (exploratory)	4,981	1.2000	1.7617	78.6	89.9
positive unreported remainder	B4 hierarchical Bayes	2,810	1.9525	3.1687	70.5	85.9
	B5 gradient boosting	2,810	1.6489	2.7447	59.9	84.1
	B8 delay-aware temporal	2,810	2.1830	3.2707	54.2	78.1
	Z1 boosted hurdle-NB (exploratory)	2,810	1.9824	2.9199	62.3	82.1
Probability that any report remains outstanding; observed event rate 7.83%						
Brier	B4 hierarchical Bayes	35,880	0.0614	—	mean predicted 14.60%	
Brier	B5 gradient boosting	35,880	0.0587	—	mean predicted 12.65%	
Brier	B8 delay-aware temporal	35,880	0.0655	—	mean predicted 12.13%	
Brier	Z1 boosted hurdle-NB (exploratory)	35,880	0.0516	—	mean predicted 9.15%	

Table S10. Zero-inflated recalibration of the frozen delay-aware predictive distributions. Cov80$^+$ is 80% coverage on cells with a positive mature target. π is the mixing weight on the point mass at zero. The final block gives paired cutoff-block contrasts with 1,000 resamples.

Model	Variant	WIS	MAE	Cov80	Cov95	Bias	Cov80$^+$
B4 hierarchical Bayes	as frozen	0.2145	0.3296	97.1	98.6	0.1782	79.1
	+ explicit zero mass	0.1741	0.2807	97.5	98.9	0.0346	82.1
B8 delay-aware temporal	as frozen	0.2294	0.3390	95.6	98.0	0.0556	68.2
	+ explicit zero mass	0.2044	0.2963	96.6	98.4	−0.0203	75.2
Fitted mixing weights, estimated on validation and frozen							
B4 hierarchical Bayes	π by age 0–3	0.253	0.280	0.388	0.459		
B8 delay-aware temporal	π by age 0–3	0.000	0.175	0.381	0.489		
Paired cutoff-block contrasts, 95% intervals on the absolute WIS difference							
B4 + zero mass vs B4		0.1741	0.2145	−0.0404 (−0.0509, −0.0308)			−18.8%
B8 + zero mass vs B8		0.2044	0.2294	−0.0249 (−0.0301, −0.0202)			−10.9%
B4 + zero mass vs B5		0.1741	0.1748	−0.0007 (−0.0071, 0.0059)			−0.4%
B8 + zero mass vs B4 + zero mass		0.2044	0.1741	0.0303 (0.0215, 0.0401)			+17.4%

7.3 Active-cohort threshold

The primary cohort requires at least two development-period outbreak-equivalents and so excludes units that report rarely. Table S11 rescores the frozen locked-test predictions at six thresholds, from the complete 360-unit panel to the 38 heaviest reporters; the cohort rule selects which units are scored and changes no model input, so no refit is involved. B5 is the best-scoring method at every threshold and its margin widens monotonically with the reporting history a unit is required to have, which is the same dependence as Table S7 measured a second way. The ordering behind B5 is not stable: the transparent comparator ranks second at the three lowest thresholds and last of the four from ≥5 upward, because the neural models gain more from restriction to high-volume units than it does.

One row deserves attention. At the most restrictive threshold B9 reaches +5.2% and would therefore have passed a 5% gate applied to those 38 units alone. That is an exploratory cohort discarding 89% of the panel, and it is the only condition under which the primary hypothesis of this study would have been supported. It is reported here rather than omitted.

Table S11. Active-cohort threshold sensitivity. The threshold is the minimum development-period matured outbreak-equivalent total required for cohort membership; * marks the prespecified primary rule.

Threshold	Units	Cells	Method	WIS	vs B4	Cov80	Cov95	Bias
≥0	360	112,320	B4 hierarchical Bayes	0.0778	—	98.8	99.4	0.0627
			B5 gradient boosting	0.0657	+15.6	97.4	99.0	0.0160
			B8 delay-aware temporal	0.0829	−6.5	98.3	99.1	0.0218
			B9 delay-aware temporal+graph	0.0824	−5.9	98.3	99.1	0.0193
≥1	136	42,432	B4 hierarchical Bayes	0.1926	—	97.3	98.7	0.1460
			B5 gradient boosting	0.1600	+16.9	93.9	97.7	0.0261
			B8 delay-aware temporal	0.2065	−7.2	95.9	98.1	0.0440
			B9 delay-aware temporal+graph	0.2056	−6.7	96.0	98.1	0.0398
≥2*	115	35,880	B4 hierarchical Bayes	0.2145	—	97.1	98.6	0.1782
			B5 gradient boosting	0.1748	+18.5	93.4	97.5	0.0275
			B8 delay-aware temporal	0.2294	−6.9	95.6	98.0	0.0556
			B9 delay-aware temporal+graph	0.2284	−6.5	95.7	98.0	0.0516
≥5	93	29,016	B4 hierarchical Bayes	0.2011	—	96.9	98.6	0.1988
			B5 gradient boosting	0.1573	+21.8	92.6	97.2	0.1072
			B8 delay-aware temporal	0.1960	+2.5	95.3	97.9	0.1034
			B9 delay-aware temporal+graph	0.1952	+2.9	95.5	98.0	0.1051
≥10	73	22,776	B4 hierarchical Bayes	0.2472	—	96.3	98.3	0.2456
			B5 gradient boosting	0.1897	+23.3	91.3	96.8	0.1315
			B8 delay-aware temporal	0.2395	+3.1	94.7	97.6	0.1278
			B9 delay-aware temporal+graph	0.2387	+3.4	94.8	97.6	0.1297
≥50	38	11,856	B4 hierarchical Bayes	0.4236	—	94.2	97.3	0.4202
			B5 gradient boosting	0.3135	+26.0	85.9	95.0	0.2025
			B8 delay-aware temporal	0.4041	+4.6	91.5	96.2	0.2035
			B9 delay-aware temporal+graph	0.4016	+5.2	91.8	96.3	0.2061

7.4 Maturity horizon

Varying the horizon required rebuilding the reporting tensor to 78 delay bins and refitting the transparent comparator, gradient boosting and Z1 at every cutoff. Here the ordering also moves: Z1 beats the comparator at 52 and 78 weeks but trails it at 26, while B5 leads at all three. The three horizons are compared over the common window of 53 cutoffs from 7 January 2024 to 5 January 2025, for which age-0 targets are mature under all three definitions, so the comparison is between horizons and not between evaluation periods. The final two columns give the share of the 78-week outbreak-equivalent target mass each horizon captures, which is what makes 52 weeks defensible: shortening the horizon costs substantially more in the slower-reporting disease than lengthening it gains in either.

7.5 Boundary week of the as-of completeness estimator

One convention was found to differ between the two systems after the test was opened, and it is reported here with its effect measured rather than described. The expanding as-of completeness estimator pools occurrence weeks whose 52-week maturity window has already closed. The WAHIS implementation excluded the single week whose window closes exactly at the cutoff; the APHIS implementation included it, as does the maturity rule (occurrence week + 52 $\leq T$) used elsewhere in the pipeline. Neither reads a report dated after the cutoff, so the exclusive form is one week more conservative rather than leaky. It nevertheless deviates from the stated inclusive rule, and the

Table S12. Maturity-horizon sensitivity; * marks the primary definition. "HPAI mass" and "ASF mass" are the percentage of the 78-week outbreak-equivalent target mass captured at that horizon.

Horizon	Method	WIS	vs B4	MAE	Cov80	HPAI mass	ASF mass
26 wk	B4 hierarchical Bayes	0.1809	—	0.2709	97.4	98.1%	91.4%
	B5 gradient boosting	0.1524	+15.8	0.2206	95.2		
	Z1 boosted hurdle-NB (exploratory)	0.1840	−1.7	0.2589	97.2		
52 wk*	B4 hierarchical Bayes	0.2026	—	0.3081	97.5	99.5%	97.7%
	B5 gradient boosting	0.1687	+16.7	0.2469	92.9		
	Z1 boosted hurdle-NB (exploratory)	0.1894	+6.5	0.2660	97.2		
78 wk	B4 hierarchical Bayes	0.2083	—	0.3171	97.5	100.0%	100.0%
	B5 gradient boosting	0.1633	+21.6	0.2327	94.1		
	Z1 boosted hurdle-NB (exploratory)	0.1916	+8.0	0.2634	97.1		

locked test ran under that exclusive implementation.

Table S13 quantifies the difference. The two transparent methods that carry the primary gate comparison were refitted on the locked test under each convention, using the same rolling-origin schedule. Refitting under the frozen convention reproduces the locked-test scores exactly, which is what licenses reading the second row as the effect of the convention alone. Aligning the boundary moves the gradient-boosting margin over the transparent comparator from +18.52% to +18.92%: the adoption decision, the direction and the order of magnitude are all unchanged. The completeness feature itself moves by 6.6×10^{-4} on average, and by more than 0.01 in 1.1% of scored cells, those being thin units whose expanding history is short enough for one extra week to matter. The frozen results reported throughout this paper are the exclusive-convention ones; the code ships with the convention named explicitly and with this check as a runnable script.

Table S13. As-of completeness boundary convention. B4 and B5 refitted on the locked test under each convention, macro-WIS over the primary active cohort and cohort ages 0–3; * marks the convention the locked test was frozen and run under. The lower block reports the shift in the completeness feature itself.

Boundary convention	B4 WIS	B5 WIS	B5 vs B4
Frozen: occurrence week + 52 $< T$ *	0.2145	0.1748	+18.52%
Aligned: occurrence week + 52 $\leq T$	0.2145	0.1739	+18.92%
Shift in the completeness feature itself, 112,320 unit × age × cutoff cells			
mean absolute shift			6.6×10^{-4}
99th percentile			0.0111
largest shift			0.3437
cells shifting by more than 0.01			1.08%

8 Simulation with known truth

Ten scenarios were simulated with 100 replicates each. The adaptive delay estimator recovered the generator's completeness more accurately than a fixed curve in nine of them, and every scenario exceeded 90% empirical coverage for nominal 95% intervals. The exception is the point of the exercise: where a single fixed curve is the true mechanism, the fixed estimator is an order of magnitude more accurate. Adaptivity buys robustness to drift at some cost in efficiency when there is no drift, and the real-data hazard tests of Table S15 are what indicate which regime applies here.

Table S14. Ten simulation scenarios, 100 replicates each. "F err" is the mean absolute error in recovered completeness. † marks the one scenario in which the fixed curve is more accurate, which is the scenario whose generator is a fixed curve.

Scenario	Adaptive F err	Fixed F err	WIS	Cov80	Cov95
S1 stable delay †	0.038	0.004	0.2975	92.9	98.9
S2 country heterogeneity	0.044	0.145	0.2826	92.9	98.9
S3 smooth drift	0.070	0.111	0.2840	93.6	99.1
S4 workload backlog	0.038	0.062	0.3393	93.2	99.0
S5 reporting changepoint	0.099	0.126	0.3423	90.6	98.1
S6 sparse, zero-heavy	0.067	0.088	0.0644	95.5	98.8
S7 long tail beyond 52 weeks	0.042	0.088	0.2724	92.9	98.9
S8 graph misspecified	0.041	0.088	0.2916	92.8	98.8
S9 cold-start units	0.044	0.088	0.2922	92.7	98.8
S10 permanently unreported	0.162	0.199	0.2326	93.2	98.9

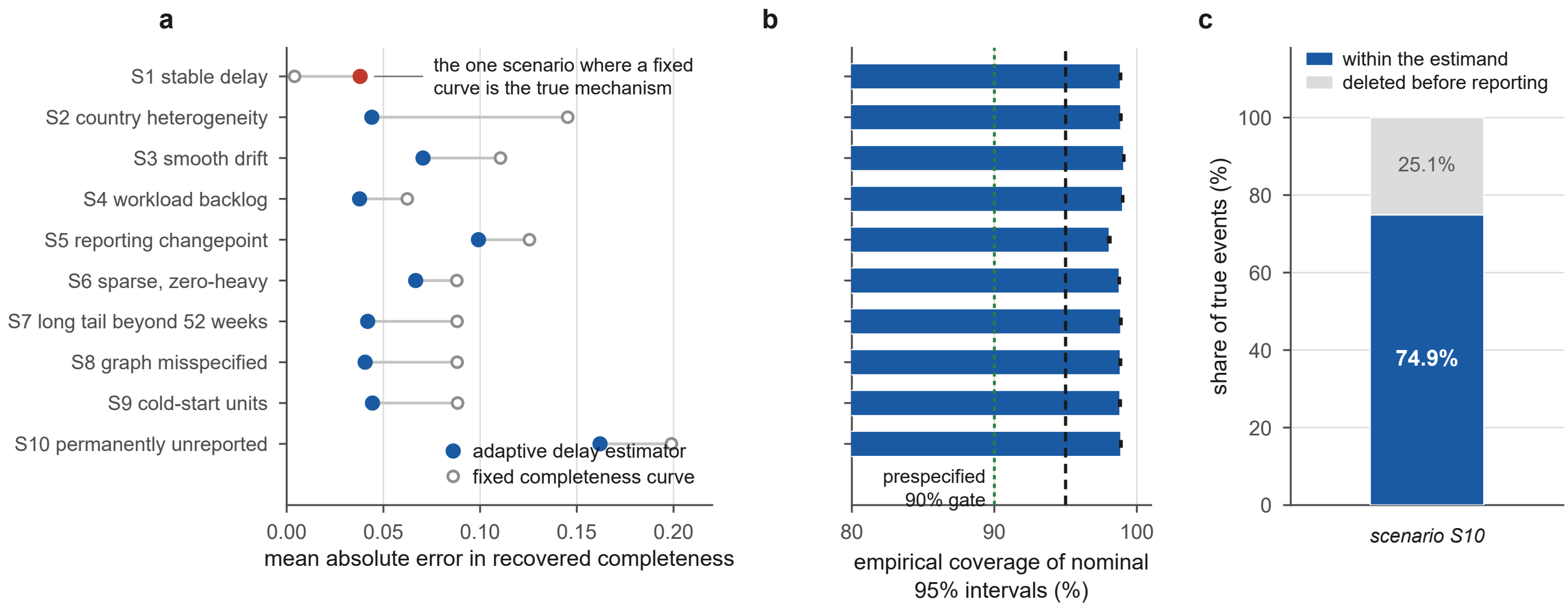

Figure S2. Ten simulation scenarios with known truth. (a) Mean absolute error in recovered completeness, adaptive estimator against a fixed completeness curve. The red marker is the one scenario in which the fixed curve wins, and its generator is a fixed curve. **(b)** Empirical coverage of nominal 95% intervals with Monte Carlo standard errors; every scenario clears the prespecified 90% gate. **(c)** The identifiability boundary in scenario S10: the estimand covers 74.9% of true events, and the 25.1% deleted before reporting are unrecoverable from the database by any method.

9 Reporting-delay heterogeneity

Nested discrete-time reporting-hazard models add host, region, year, season and recent load in sequence. Every addition reduced deviance in both diseases. With samples of this size the p-values are read as evidence of heterogeneity rather than as a measure of magnitude; the AIC changes and the visibly separated completeness curves are what support estimating completeness adaptively instead of from one pooled curve.

Table S15. Nested discrete-time reporting-hazard models. Δdeviance and p compare each model with the one above it.

Disease	Model	Deviance	AIC	Δdeviance	df	p
HPAI	delay only	123139.3	132596.7	—	—	—
	+ host	122863.5	132322.9	275.78	1	6.2×10^{-62}
	+ region	118834.8	128304.3	4028.64	5	$< 10^{-99}$
	+ year	118362.0	127833.4	472.87	1	$< 10^{-99}$
	+ season	117846.4	127321.8	515.57	2	$< 10^{-99}$
	+ recent load	117436.9	126914.3	409.54	1	4.6×10^{-91}
ASF	delay only	344595.4	355425.6	—	—	—
	+ host	343082.1	353914.3	1513.31	1	$< 10^{-99}$
	+ region	336817.3	347655.5	6264.80	3	$< 10^{-99}$
	+ year	336793.2	347633.3	24.15	1	8.9×10^{-07}
	+ season	335585.1	346429.3	1208.06	2	$< 10^{-99}$
	+ recent load	334763.8	345610.0	821.26	1	$< 10^{-99}$

10 Cluster handling, target definition and host exclusion

Rows representing a declared cluster were expanded by the reported number of outbreaks for the primary target. Table S16 repeats the locked evaluation under the two alternatives. Neither reverses any conclusion.

Table S16. Cluster handling and target definition, HPAI locked test.

Target definition	Method	WIS	vs B4	Cov80	Bias
cluster-expanded (primary)	B4 hierarchical Bayes	0.2145	—	97.1	0.1782
	B5 gradient boosting	0.1748	+18.5	93.4	0.0275
	B6 temporal, no delay	0.2762	−28.8	97.9	0.0943
	B8 delay-aware temporal	0.2294	−6.9	95.6	0.0556
clusters excluded	B4 hierarchical Bayes	0.2146	—	97.1	0.1823
	B5 gradient boosting	0.1748	+18.5	93.4	0.0316
	B6 temporal, no delay	0.2729	−27.2	97.9	0.0984
	B8 delay-aware temporal	0.2294	−6.9	95.6	0.0597

The mammalian-host exclusion is reported separately because how it was computed changed the answer. The 1,543 mammalian-host HPAI outbreaks are 4.0% of retained HPAI outbreaks but 19.2% of the matured locked-test target mass (1,028 of 5,360 outbreak-equivalents), affecting 0.81% of scored unit-weeks . Recomputing the target, rescoring the stored predictions and skipping the refit produced a sign reversal (+10.9% against −13.7%) and dropped the rank correlation of model scores from 0.993 to 0.70. Models fitted to a target containing a large, bursty, geographically concentrated component are handicapped when scored against a target without it, most of all where the observed count is used most aggressively. A full rebuild and refit removed the artefact entirely. The same principle was therefore used for the maturity-horizon analysis, where redefining the target invalidates stored predictions; the cohort-threshold analysis instead retains the target and appropriately re-aggregates the frozen predictions.

11 Lead time

Lead time is measured against an official report becoming visible, not against laboratory confirmation and not against true infection. A unit-week is flagged when the estimated probability of an outstanding report exceeds 0.5.

12 Hyperparameter search

Sixteen configurations were fitted on validation data. The selection score is validation macro-WIS with a calibration penalty; lower is better. The search budget was reduced relative to the protocol, with each trial fitted once at

Table S17. Lead time relative to the first visible official report, HPAI locked test.

Method	Unit-weeks flagged	Preceded first report	Median lead	Mean lead
B4 hierarchical Bayes	957	64.7%	1.0 wk	1.45 wk
B5 gradient boosting	858	74.6%	1.0 wk	1.59 wk
B8 delay-aware temporal	1,095	36.0%	0.0 wk	0.74 wk
B9 delay-aware temporal+graph	1,097	35.8%	0.0 wk	0.74 wk

the first validation refit point rather than refitted across all cutoffs, and that deviation is recorded in the frozen protocol.

Table S18. Hyperparameter search on validation data, ordered by selection score. * marks the configuration frozen for the locked test.

Trial	Encoder	d	Layers	Dropout	LR	Params	WIS	Score
3*	transformer	32	2	0.2	0.001	24,229	0.4547	0.5100
13	tcn	64	2	0.1	0.001	39,429	0.4578	0.5129
11	tcn	32	2	0.1	0.001	12,549	0.4580	0.5133
15	tcn	64	3	0.1	0.001	55,941	0.4613	0.5174
4	transformer	32	3	0.1	0.0003	32,773	0.4641	0.5221
2	transformer	32	2	0.2	0.0003	24,229	0.4658	0.5245
1	transformer	32	2	0.1	0.0003	24,229	0.4661	0.5247
12	tcn	64	2	0.1	0.0003	39,429	0.4677	0.5252
10	tcn	32	2	0.1	0.0003	12,549	0.4678	0.5252
9	transformer	64	3	0.2	0.001	118,789	0.4730	0.5296
8	transformer	64	3	0.1	0.001	118,789	0.4755	0.5319
5	transformer	64	2	0.1	0.001	85,317	0.4768	0.5336
6	transformer	64	2	0.2	0.0003	85,317	0.4777	0.5363
7	transformer	64	3	0.1	0.0003	118,789	0.4785	0.5369
16	tcn	64	3	0.2	0.0003	55,941	0.4858	0.5456
14	tcn	64	3	0.1	0.0003	55,941	0.4858	0.5456

References cited in this Supplementary Material

Bai, S., Kolter, J.Z., Koltun, V., 2018. An empirical evaluation of generic convolutional and recurrent networks for sequence modeling. https://arxiv.org/abs/1803.01271. doi:10.48550/arXiv.1803.01271, arXiv:1803.01271.

Mullahy, J., 1986. Specification and testing of some modified count data models. Journal of Econometrics 33, 341–365. doi:10.1016/0304-4076(86)90002-3.

Schlichtkrull, M., Kipf, T.N., Bloem, P., van den Berg, R., Titov, I., Welling, M., 2018. Modeling relational data with graph convolutional networks, in: The Semantic Web (ESWC 2018), Springer International Publishing, Cham. pp. 593–607. doi:10.1007/978-3-319-93417-4_38.

Vaswani, A., Shazeer, N., Parmar, N., Uszkoreit, J., Jones, L., Gomez, A.N., Kaiser, Ł., Polosukhin, I., 2017. Attention is all you need, in: Advances in Neural Information Processing Systems, Curran Associates, Inc.. pp. 5998–6008. ArXiv:1706.03762.
